# Global Prevalence of Perinatal Depression and Its Determinants Among Rural Women: A Systematic Review and Meta-Analysis

**DOI:** 10.1155/2024/1882604

**Published:** 2024-09-20

**Authors:** Ting Pan, Yi Zeng, Xiaoni Chai, Zhang Wen, Xiangmin Tan, Mei Sun

**Affiliations:** ^1^Xiangya School of Nursing, Central South University, Changsha 410013, China; ^2^School of Nursing, Changsha Medical University, Changsha 410219, China; ^3^International School of Nursing, Hainan Vocational University of Science and Technology, Haikou 525028, China; ^4^Xinqiao Hospital, Army Medical University, Chongqing 400037, China; ^5^School of Rural Health, Monash University, 15 Sargeant Street, Warragul, Victoria 3820, Australia; ^6^School of Nursing, Xinjiang Medical University, Urumqi 830054, China

## Abstract

**Background:** Perinatal depression (PND) in low-resource areas is a significant concern that imposes a substantial burden on both families and societies. Although many studies have explored rural PND, there is a lack of systematic synthesis of the existing research. This study aimed to estimate the global prevalence of PND among rural women and to summarize its determinants.

**Methods:** Comprehensively electronic searches were performed across eight English databases. Two reviewers independently assessed the eligibility of the study and extracted the relevant data. Any inconsistencies were resolved through discussion with a third reviewer. Prevalence estimates were calculated using a random-effects meta-analysis model. Subgroup analysis, sensitivity analysis, and meta-regression were employed to examine the source of heterogeneity. In addition, a narrative synthesis of the influence factors reported in the included studies was provided.

**Results:** The search identified 17,810 studies, of which 86 were included in the analyses. The pooled prevalence of PND in rural areas was 22.1% (95% CI 19.0%–25.3%, *p* < 0.001, *I*^2^ = 99.2%). Subgroup analyses indicated higher PND prevalence in low-income (24.5%) and lower middle-income countries (22.8%). Additionally, PND prevalence was greater when assessed using self-reported screening instruments (22.8%) compared to diagnostic interviews (17.6%). Major risk factors included violence, antenatal psychiatric disorder, low family income, male-child preference, and food insecurity, while positive social support and higher levels of education were protective factors.

**Conclusions:** Our findings suggest that the prevalence of PND is higher in rural areas compared to global data, particularly in low-income and lower middle-income countries. To improve rural maternal mental health, it is essential to develop measures targeting modifiable risk factors for PND, including promoting gender equality, implementing antiviolence initiatives, and strengthening economic support systems. Addressing these factors can help reduce the burden of PND and enhance the well-being of mothers in rural communities.

## 1. Introduction

Perinatal depression (PND) refers to the occurrence of a depressive episode in a woman during pregnancy and within the first year after giving birth [1]. It is one of the most common medical complications during pregnancy and postpartum, which includes major and minor depressive episodes [2]. The global burden of PND is 11.9%, with a higher prevalence in low- and middle-income countries (13.1%) than in high-income countries (11.4%) [3]. PND has a great negative impact on individuals and society. It is associated with premature labor, preeclampsia, low birth weight, infanticide, and suicide [4, 5]. In addition, maternal depression may lead to insecure attachment with the child, potentially affecting the child's development in areas, such as cognitive, language, motor, and adaptive behaviors [6].

The WHO suggests that social inequality is closely linked to risk factors for many common mental disorders. The greater the inequality, the higher the inequality in risk [7]. The urban–rural divide is a universal phenomenon, prevalent in all regions and countries [8]. Living in rural areas may negatively impact people with mental disorders. Rural residents face limited access to professional mental health services, primarily due to a shortage of mental health professionals and geographical barriers [9]. The mental health knowledge gap between urban and rural areas often results in rural residents lacking awareness of mental disorders, which reduces their likelihood of seeking help [10]. Moreover, the prevalent stigma surrounding mental disorders in rural areas, combined with the lack of anonymity, prompts individuals to conceal their mental disorders, hindering proper identification, treatment, and management of such problems [10, 11]. Evidence showed that suicide rates due to mental illness in rural areas are often higher than in urban areas [12].

Identified risk factors for PND include intimate partner violence, poverty, inadequate social support, and limited access to maternal health services [13], which are more prevalent in rural areas [8, 14]. Combined with limited treatment options, women living in rural areas may be at higher risk of developing PND. Some studies have found that living in rural areas is a risk factor for PND [15–17]. Additionally, rural women with PND are less likely to be well-diagnosed and treated due to the low accessibility, availability, and acceptability of mental health services, which can result in a significant social burden. In the US alone, untreated perinatal mental health issues incur a $14 billion annual social burden [18]. A study comparing healthcare utilization and costs for PND in the US found that rural residents had more hospital days and emergency department visits, and spent more on inpatient services compared to urban residents ($2,654 vs. $1,786) [19].

A review based on 17 studies found the prevalence of postpartum depression among rural women to be 27.0%, with a higher prevalence observed in women from developing countries compared to those from developed countries [20]. To our knowledge, this is the only systematic review and meta-analysis analyzing the global prevalence of postpartum depression in rural areas. However, this review is limited by its inclusion of a relatively small number of studies and its exclusion of pregnant women. In addition, previously reported global prevalence of PND was based primarily on studies conducted in urban areas. For instance, a systematic review and meta-analysis of postpartum depression included 54 studies, with 46 of those studies focusing on urban populations [21]. These research gaps underscore the need to address two critical questions: What is the global prevalence of PND in rural areas? And what factors contribute to PND in rural areas?

A comprehensive evaluation of the prevalence of PND in rural areas globally, along with the early identification of its determinants, will help alleviate extreme behaviors such as suicide and self-harm caused by depression. This approach will not only improve the physical and mental health of rural women and promote the healthy growth and development of infants but will also reduce the social burden caused by PND. Furthermore, global estimates of rural PND prevalence are critical to understanding its global distribution and data availability. This estimate could provide valuable reference information and data support for policy formulation and resource allocation in countries that neglect perinatal mental health in rural areas. Consequently, this review aims to estimate the prevalence of PND among rural women and to further summarize the factors influencing rural PND.

## 2. Methods

The meta-analysis was registered with PROSPERO (Identifier CRD42023427648) and reported using preferred reporting items for systematic reviews and meta-analysis (PRISMA) guidelines [22].

### 2.1. Research Strategy

We systematically searched eight English databases, including PubMed, Web of Science, Cochrane Library, EMBASE, CINAHL, PsycINFO, Scopus, and Joanna Briggs Institute (JBI) Evidence-Based Practice Database. For a comprehensive data collection, we searched the entire literature from inception to September 10, 2023. We used “perinatal,” “depression,” and “rural” as key concepts. Keywords or mesh terms used include (depression OR depressive disorder OR melancholia) AND (peripartum period OR perinatal OR peripartum OR postpartum OR postnatal OR childbirth OR puerperal OR parturition OR maternal OR puerper*⁣*^*∗*^ OR postbirth OR after birth OR mother*⁣*^*∗*^ OR intrapartum OR prenatal OR antepartum OR antenatal OR pregnan*⁣*^*∗*^) AND (rural population OR rural area OR rural*⁣*^*∗*^ OR remote*⁣*^*∗*^ OR village OR hamlet OR countryside OR rustic*⁣*^*∗*^). For specific search strategies, please see Supporting Information S1: File 1. Additionally, we manually searched reference lists of included studies and grey literature (OpenGrey and PsycEXTRA) to locate more records.

### 2.2. Inclusion and Exclusion Criteria

Inclusion criteria are as follows: (1) study population consisted of women residing in rural areas who were pregnant or within 12 months postpartum; (2) study reported the prevalence of depression during pregnancy or the postpartum period or provided data that allowed estimation of its prevalence; (3) assessment of PND using diagnostic interviews or validated self-reported screening instruments, such as Edinburgh Postnatal Depression Scale (EPDS) [23]; and (4) study types included longitudinal cohort studies, cross-sectional studies, randomized controlled trials (only baseline data). It is worth noting that we included studies as long as they indicated that it is conducted in rural contexts, given the lack of a universally standardized definition of “rural” across all nations [24].

Exclusion criteria are as follows: (1) not published in English; (2) case study, editorial, guideline, and review article; and (3) studies targeting specific populations (e.g., HIV-infected women, adolescents). These conditions may represent different risks for PND, and the inclusion of these populations would limit the generalizability [25].

### 2.3. Study Selection

**Figure 1:**
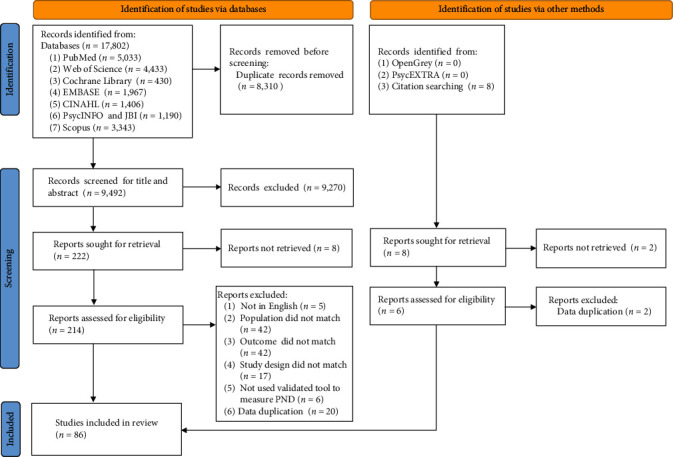
Preferred reporting items for systematic reviews and meta-analyses (PRISMA) flow diagram for the study selection process.

Two reviewers independently screened titles, and abstracts and conducted full-text reviews. Disagreements were resolved through discussion with a third reviewer. The screening and full-text review were performed using Endnote 20.0 software. The screening process is shown in Figure 1.

### 2.4. Data Extraction

Two reviewers independently extracted data using a unified Excel table from the eligible studies as follows: name of the first author, year of publication, study location, setting, study design, sampling method, sample size, measurement of depression, cut-off points, gestational period (antenatal or postnatal), identified cases, and prevalence of depression. If multiple data sources were reported on the same sample, a more informative data source was used. For studies with multiple time points of depression, we extracted antenatal and postnatal data separately for the one closest to the time of birth. We excluded the first 2 weeks after birth to account for the postpartum blues [26]. For studies that reported separately the prevalence of minor and major PND, data were obtained by dividing the number of women with minor and major PND by the total number of women in the study and multiplying by 100%. Any disagreements were resolved by discussion with the third reviewer. To ascertain risk factors for PND among rural women, we enumerated the specific risk factors for narrative synthesis.

### 2.5. Quality Assessment

Quality assessment for eligible studies was conducted by two reviewers independently. Any disagreements were discussed and consulted with the third one. We assessed the quality of cross-sectional studies, longitudinal cohort studies, and randomized controlled trials using the 11-item instrument recommended by the Agency for Healthcare Research and Quality (AHRQ) [27], the Newcastle–Ottawa Scale (NOS) [28], and the Modified Jadad Scale [29], respectively. The AHRQ is scored out of 11 using “yes,” “no,” or “not sure” to answer each item, with a total score of 0–3 as low quality, 4–7 as moderate quality, and 8–11 as high quality. The NOS includes three dimensions: selection, comparability, and outcome. Studies with total scores ranging from 0 to 3, 4–6, and 7–9 were considered low, moderate, and high quality, respectively. The Modified Jadad Scale evaluates the quality of randomized controlled trials in terms of four dimensions: randomization, allocation concealment, blinding, and dropout, with a total score of 4–7 representing high quality and a total score of 0–3 representing low quality.

### 2.6. Data Analysis

All statistical analysis was performed with Stata MP 17. Prevalence estimates were extracted as raw proportions. Two data points with a prevalence of 0% were not included in the analysis. Other potential outliers were retained in the dataset to maximize inclusion. Pooled estimates were calculated using random effects meta-analysis based on geographic heterogeneity and variability in screening and diagnostic tools [30] and displayed in forest plots. Statistical heterogeneity was estimated using the *I*^2^ statistic. Potential sources of heterogeneity were investigated through subgroup analyses and sensitivity analyses, including the exclusion of studies with prevalence estimates below 5% and above 60% [31], and those evaluated as low quality. Subgroup analyses were performed according to gestational period, setting, region, country income group (the World Bank Classification) [32], study design, sampling method, and assessment instrument. Meta-regression with random effect and the maximum likelihood method was employed to examine the source of heterogeneity, which could determine whether prevalence estimates were conditional on certain moderators. Publication bias was assessed by funnel plot and Egger'slinear regression test [33]. All *p* values are < 0.05 suggesting statistical differences for all analyses. Moreover, the included studies did not uniformly assess the relationship between PND and other covariates, precluding meta-analyses. Therefore, we provide a narrative summary of the determinants of PND.

## 3. Results

### 3.1. Search Results

The systematic search yielded 17,802 total studies. After removing duplicates and reviewing the titles and abstracts, we identified 214 potentially relevant studies. Following a full-text review, 132 were excluded based on the inclusion criteria. Additionally, four studies were obtained by searching the references of the included studies. Finally, 86 studies were included in the meta-analysis, including 84 observational studies (22 longitudinal studies and 62 cross-sectional studies) and two randomized controlled trials (see Figure 1 for details).

### 3.2. Characteristics of Included Studies

The 86 eligible studies from 28 countries were published between 2002 and 2023, with India contributing the most studies (*n* = 20), followed by Bangladesh (*n* = 8), China (*n* = 7), America (*n* = 6), and Ethiopia (*n* = 6). According to the World Bank's most recent classification of country incomes [32], 12 studies were identified from high-income countries, 11 studies from upper middle-income countries, 52 studies from lower middle-income countries, and 11 studies from low-income countries. Eighty-six studies yielded a total of 101 data points, which reported postpartum depression (*n* = 53), antenatal depression (*n* = 43), and PND (*n* = 5). Most of the data used nonrandom sampling techniques (*n* = 81) instead of random sampling (*n* = 20). Fifty-six data were collected in a community or primary health setting, 21 in a hospital setting, six were collected across multiple settings and another 18 data were population-based. Eighty-seven data used self-reported screening instruments to determine the prevalence, while 14 used diagnostic interviews. The most frequently used instrument was EPDS (*n* = 54). For data using identical self-reported screening instruments to identify depressive symptoms, there were variations in the cut-off values among different data. For example, the cut-off score for depressive symptoms ranged from 9 to 13 on the EPDS. In addition, impact factors were reported in 32 studies. Scores of methodological qualities of included studies ranged from moderate to high quality. Details of the included studies are shown in Supporting Information S2: File 2.

### 3.3. Prevalence of PND

**Figure 2:**
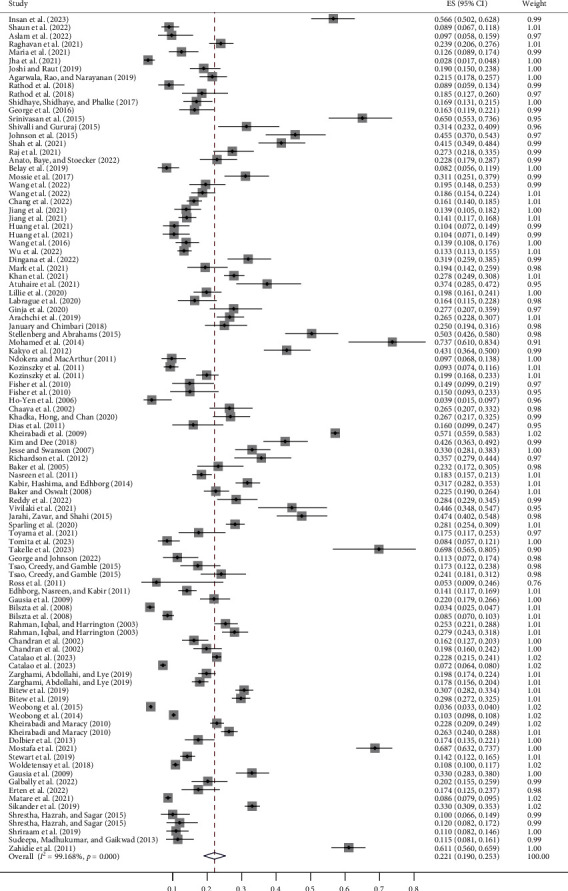
Forest plot of the prevalence of perinatal depression.

The pooled prevalence estimate of any PND across 86 studies with 101 data points was 22.1% (95% CI 19.0%–25.3%, *p* < 0.001, *I*^2^ = 99.2%). See Figure 2 for a detailed forest plot. The prevalence of PND exhibited notable variations contingent upon factors such as study design, sampling method, gestational period, country income group, setting, region, and the assessment instrument employed. The pooled prevalence of antenatal depression was 23.3% (95% CI 19.7%–27.2%), while postpartum depression was significantly lower at 21.1% (95% CI 15.8%–27.0%). Five studies combined antenatal and postnatal women and reported a prevalence of depression of 21.0% (95% CI 14.5%−28.4%). In terms of country income groups, the prevalence was highest in low-income countries at 24.5% (95% CI 18.3%–31.3%) and lowest in upper middle-income countries at 17.9% (95% CI 14.7%–21.3%). The prevalence varied substantially among countries, from 10.1% (Ghana, 95% CI 4.4%–17.8%) to 33.6% (Pakistan, 95% CI 25.4%–44.3%). The prevalence of PND identified through the diagnostic interview (17.6%, 95% CI 12.7%–23.1%) was lower than the self-reported screening instruments (22.8%, 95% CI 19.4%–26.4%). Of the different self-reported screening instruments, the prevalence identified by the Beck Depression Inventory was the highest (36.1%, 95% CI 20.3%–53.7%), followed by the EPDS (22.4%, 95% CI 19.2%–25.8%). Additionally, prevalence estimates were highest among studies performed in multiple settings (32.6%, 95% CI 19.9%–46.7%) and lowest in population-based studies (15.1%, 95% CI 10.9%–19.7%) (see Table 1 for more details).

### 3.4. Sensitivity Analysis and Publication Bias

**Figure 3:**
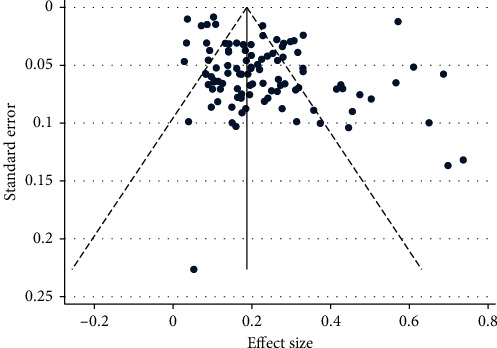
Funnel plot of studies.

Sensitivity analysis by removing studies one by one did not reveal any significant changes in prevalence rates of PND. We excluded eight data with prevalence rates below 5% or above 60%, but the effect on the point estimate was negligible. The pooled prevalence estimate from the remaining studies was 20.9% (95% CI 18.0%–23.9%, *p* < 0.001, *I*^2^ = 98.9%). According to the visual inspection of the funnel plot (Figure 3), publication bias was shown in the included studies, which is consistent with the results of Egger's test (*t* = 2.21, *p*=0.029). After using the trim and fill method with the random effects model, there was no variation in adjusted effect sizes.

### 3.5. Meta-Regression

Significant heterogeneity was evident across all studies included in the analysis. A meta-regression was performed by setting, country income group, study design, sampling method, assessment instrument, region, and gestational period to explain the heterogeneity source. The findings indicated that the study design exerted a discernible impact on the estimated prevalence of PND (*p* < 0.05), as detailed in Table 2.

### 3.6. Risk Factors of PND

Significant risk factors of PND were reviewed systematically and divided into four categories: (1) demographic factors; (2) psychosocial factors; (3) pregnancy-related and infant-related factors; and (4) other factors.

#### 3.6.1. Demographic Factors

In rural areas, younger (up to 25 years old) and older (35 years and above) women [34–36], particularly those with lower incomes, are at a higher risk of developing PND [37, 38]. Marital status has also been linked to PND, with unmarried women more frequently reported to experience PND compared to married counterparts across several studies [37, 39, 40]. Educational attainment and employment status are additional factors scrutinized with PND among rural women. Most studies underscore that unemployed or less educated rural women are disproportionately affected by PND [34, 35, 37, 41–44]. However, the two studies have shown divergent trends, indicating higher PND rates among skilled or professional women [39], and those with higher educational achievements [45]. In addition, the employment status of the husband appears to be an important factor in rural maternal mental health. One study found that rural women with unemployed husbands faced a 4.4 times higher risk of postpartum depression compared to those whose husbands were employed [46]. Studies examining family structure have revealed varying impacts on PND. Research indicates a higher prevalence of PND among women in joint families, extended families, or residing with their parents [41, 47, 48]. Conversely, findings from Pakistan suggest that women in a nuclear family may face an increased risk of postpartum depression [46]. Moreover, the number of children in the household appears to play a role, with both having only one child [43] or more than two children [45] associated with heightened PND risk.

#### 3.6.2. Psychosocial Factors

Some of the included studies suggest that PND in rural areas is frequently influenced by a complex interplay of psychosocial factors. Insufficient social support during the perinatal period emerges as a critical determinant, encompassing both tangible support deficits [38] and a lack of care from the biological mother [49], significantly heightening the vulnerability of women to PND. Moreover, the cultural pressure favoring male offspring in rural regions of certain low- and middle-income countries cannot be ignored. On the one hand, the perceived expectations from husbands or families for a male child impose substantial psychological stress on pregnant women, thereby increasing their risk of antenatal depression [40, 50, 51]. On the other hand, mismatches between the gender of the child and familial expectations are associated with heightened trauma and an elevated risk of postpartum depression [34, 38, 52]. The issue of intimate partner violence is equally pressing. Whether occurring before, during, or after pregnancy, intimate partner violence—encompassing psychological, physical, and sexual abuse—severely undermines women's mental well-being and contributes to depression [36, 48, 50, 53–58]. Unharmonious family relations, such as poor relations with husbands, in-laws, and parents, and conflicts within the family exacerbate women's depression [36, 38, 40, 46, 51, 58–61]. A history of mental illness is another pivotal factor. Previous psychiatric conditions in women, particularly depression, suicidal tendencies, and familial history of psychiatric disorders, elevate the likelihood of PND [34–36, 49, 55, 56, 60]. Other psychosocial factors associated with PND in women include higher stress, lower self-esteem, less spiritual perspective [54], lower sense of hope [53], dissatisfaction with life [48], concerns regarding the health of the baby [49], adverse life events during pregnancy [38], and exposure to multiple adverse life events [48].

#### 3.6.3. Pregnancy-Related and Infant-Related Factors

Some of the included studies reported the relationship between pregnancy-related factors and PND in rural areas. Unplanned pregnancy emerges as a notable risk factor as it may induce psychological stress and uncertainty for women, consequently increasing the risk of depression [34, 35]. Additionally, a history of abortion and unfortunate experiences such as perinatal death may cause deep psychological trauma to women and increase their likelihood of developing depressive symptoms during the perinatal period [40, 45, 59, 60]. Complications during pregnancy and childbirth also contribute to the risk of depression in women [45, 52]. In addition, several studies have identified certain infant-related factors as important risk factors for PND, including low birth weight [57, 62], acute infant respiratory infections [62], male infant gender [56], and having a fussy and demanding infant [58].

#### 3.6.4. Other Factors

Other factors related to PND in rural areas include socioeconomic and physiological factors. Socioeconomic factors such as household economic hardship or poverty often increase the risk of depression among rural perinatal women [40, 47, 52, 56, 59]. Notably, five studies have identified food insecurity as a risk factor for PND [53–63]: higher levels of food insecurity correlate with increased PND risk. Additionally, physiological factors also play a crucial role. Anemia, physical illness, multiple medical diseases, and irregular sleep patterns were associated with PND in a single study [41, 45, 55, 59].

### 3.7. Protective Factors of PND

Six studies reported that good social support before and after childbirth serves as a potent protective factor against PND. This support encompasses practical material and emotional support from family, friends, and significant others [46, 48, 53, 55, 61], with specific emphasis on daily family support for childcare [46]. Besides, perceived good physical health status [64], vaginal delivery [43], higher educational attainment [36, 47], suitable living conditions [48], enhanced decision-making autonomy [61], higher education level of husband [45], higher socioeconomic status [65], and prenatal folic acid intake [47] have been identified as protective factors against PND.

## 4. Discussion

This systematic review and meta-analysis estimated the prevalence of PND among rural women and summarized its determinants. Eighty-six studies were included in this meta-analysis. The pooled prevalence of PND in rural areas was 22.1%, with 23.3% pooled prevalence in the antenatal period and 21.1% in the postnatal period. Variations in the prevalence of PND in rural areas were observed across different country income groups, study designs, sampling methods, settings, regions, and assessment instruments employed. The most common modifiable risk factors of rural PND included violence, low family income, male-child preference, and food insecurity. Positive social support and education emerged as prevalent protective factors against PND.

The pooled prevalence of PND in rural areas from this meta-analysis was 22.1%, which notably exceeds the previously reported global estimate of 11.9% [3]. This discrepancy can largely be attributed to the distinct demographic focus of the studies included in our analysis, which concentrated exclusively on rural populations, unlike the predominantly urban participants in global studies. Social determinants may explain high PND prevalence in rural areas: First, poverty is more prevalent in rural areas. The social conditions of poverty, including low education, low socioeconomic status, and inadequate food and housing, increase the risk of mental illness in rural populations, especially in low- and middle-income countries [66]. Second, gender inequality is starkly pronounced in rural areas, where traditional patriarchal structures often grant men predominant household authority, leaving women economically dependent and susceptible to mental health challenges [67]. Gender-based violence further compounds these risks, significantly increasing the likelihood of PND among rural women [68]. Third, the generally lower educational level of rural women may hinder their understanding and recognition, resulting in self-stigma and reluctance to seek professional help [69]. Moreover, the limited mental health literacy in rural areas has fostered widespread negative stereotypes of individuals with mental illness [70], further deterring women from seeking necessary psychosocial support [71]. Fourth, geographical and economic constraints restrict healthcare access in rural areas, posing formidable barriers for perinatal women in need of essential health services [15, 50]. This unequal distribution of resources may hinder women from receiving prompt and effective care when facing issues or emotional distress.

The studies included in our review were from only 28 countries, mostly from Asia and Africa. India contributed the largest volume of data, which aligns with the United Nations' findings that Asia and Africa collectively harbor 90% of the global rural population, with India specifically hosting the largest rural populace [72]. This demographic skew significantly influenced our findings. Besides, there is a lack of data on the prevalence of rural PND in many countries and regions. This may be due to the absence of a clear definition of rural in some countries, hindering research [24]. Inadequate infrastructure for data collection and a shortage of trained personnel further impede comprehensive data-gathering efforts in rural areas. Additionally, mental health in rural areas is often neglected by certain countries, leading to insufficient policy support and financial resources allocated to mental health research and interventions. Overall, the results of the pooled meta-analyses should be treated with caution due to the small number and uneven distribution of countries included.

In this meta-analysis, the pooled prevalence of PND in rural areas varies across different socioeconomic levels of countries. Higher prevalence rates were observed in rural areas of low-income (24.5%) and lower middle-income (22.8%) countries, consistent with prior studies [73, 74]. This may be because maternal women in high-income and upper middle-income countries with high-quality medical resources, which allows for greater access to screening and treatment for depression. It has been reported that up to 80%–90% of individuals with depression in low- and middle-income countries remain unidentified or untreated, compared to approximately 50% in higher-income countries [75]. Moreover, economic growth and increased internet accessibility in higher income countries have contributed to heightened awareness of PND, reducing the stigma associated with mental illness. Consequently, mothers with depressive symptoms are more likely to seek psychological services as the stigma of mental illness decreases. It is worth noting that the prevalence of rural PND varies even among countries with similar economic classifications. For example, among low- and middle-income countries, it is 25.4% in Bangladesh and 19.4% in India. To gain a clearer understanding of the prevalence of rural PND, countries should strengthen relevant research and surveillance, and take appropriate interventions to reduce the adverse impacts of PND in rural areas.

In addition, a systematic review and meta-analysis found that the overall prevalence of postpartum depression based on diagnostic interviews was 12.1%, which is lower compared to the prevalence largely based on self-report screening instruments [21]. We found a similar result. Screening instruments often overestimate prevalence rates due to they tend to prioritize sensitivity over specificity [76]. Throughout our meta-analysis, over 86% of the studies employed self-reported screening instruments to identify PND, with the EPDS being the primary instrument. While the EPDS total score correlates with depression severity, it is designed primarily as a screening rather than a diagnostic tool [77]. The diagnosis of PND requires a skilled clinician to make a comprehensive judgment about the patient based on their medical history, lab tests, psychological evaluations, and a diagnostic interview [77]. Unfortunately, conducting diagnostic interviews in rural areas is often impractical due to limited resources and access to trained professionals. Given these constraints, self-report screening instruments remain more feasible in low-resource settings like rural areas. However, where circumstances allow, combining self-reported screening with diagnostic interviews could potentially reduce diagnostic errors and enhance the accuracy of PND diagnosis.

The majority of risk factors for PND such as violence, antenatal psychiatric disorder, low family income, and male-child preference in this review align closely with findings from previous studies [73, 78]. However, a notable difference is the identification of food insecurity as a risk factor in several studies, all of which were conducted in low-income and lower middle-income countries. Research has shown that poorer mental health is often associated with climate extremes and biodegradable additives in rural areas [79]. Agricultural populations, common in low- and middle-income countries, are particularly vulnerable to these challenges [80]. In addition, gender inequality in low- and middle-income countries exacerbates food insecurity among rural women. Women tend to own less land and have less economic autonomy than men [81]. Cultural norms and patriarchal systems in some regions restrict women's access to adequate food resources [81]. Furthermore, women's societal roles of caring for children and the elderly often lead them to prioritize their family members' food needs over their own, especially during food scarcity [81]. These factors contribute to the lack of food security for rural women. The United Nations Women [82] predict that nearly a quarter of women will experience moderate or severe food insecurity by 2030 if gender inequality is not effectively addressed. Governments must prioritize food production, protection, and storage strategies in rural areas, as well as take action to address gender disparities to empower women globally and enhance maternal mental health.

Several demographic risk factors associated with PND (such as family structure and employment status) in rural areas exhibit varied findings across the reviewed studies. The differences highlight the nuanced relationship between demographic factors and PND, emphasizing its complexity and lack of absolute correlation. Family structure and interaction patterns are often linked to the social support accessible to the individual. Studies in Turkey have indicated that interpersonal relationships and social support, rather than family structure, are the primary factors influencing maternal depression [83, 84]. The impact of employment status on PND is also complex. Professional work can offer women economic independence, social recognition, and personal fulfillment, potentially benefiting mental health [85]. However, challenging work conditions such as excessive workloads, inflexible hours, and inadequate support and resources may heighten stress levels, thereby increasing vulnerability to PND [86]. More importantly, in some rural areas, employed women face the significant expectation of balancing various responsibilities. Alongside their paid employment, they are also required to take on unpaid domestic work, child-rearing, and caregiving for family members. These multiple burdens result in a high degree of dispersion of time, energy, and resources, making it difficult for them to respond effectively to the challenges and pressures of life. It is crucial to acknowledge that the pressures experienced by women are not solely attributed to their workload and familial responsibilities but are also influenced by societal stereotypes and gender inequalities. Therefore, when exploring the impact of demographic factors on PND in rural contexts, it becomes imperative to delve beyond surface-level factors and instead scrutinize underlying social structures, cultural contexts, and gender dynamics associated with these factors. This approach is essential for comprehensively understanding the multifaceted nature of PND in rural areas and for devising effective interventions and support systems tailored to address these complexities.

This review also has limitations. First, there is significant heterogeneity among the included studies, potentially leading to publication bias and confounding factors that could compromise the accuracy and reliability of findings. The main reasons for this may be differences in study design. Differences in research duration, samples, and outcome assessments may also be potential influences on heterogeneity. Future meta-analysis studies should consider these factors during the design phase to minimize heterogeneity and enhance the precision and robustness of results. Second, associations between PND and other factors were not uniformly assessed in the included studies, precluding meta-analysis. Our study provided only a narrative review of the determinants of PND, limiting deeper insights into the distinct impacts of each risk and protective factor on PND. Third, the majority of studies included in our review were conducted in lower middle-income countries, with fewer representations from other income categories. This imbalance may be attributed to our inclusion criteria focusing solely on English-language publications, potentially overlooking relevant studies published in other languages and thereby affecting the comprehensiveness of our review.

Improving maternal health is a key concern of the Sustainable Development Goals [87], and perinatal mental health is a severely underestimated determinant [13]. Despite increasing global awareness of PND, it remains neglected in resource-poor rural areas. It is important to clarify the direction of maternal mental health care in rural areas. This review has implications for policy, research, and clinical practice. First, insufficient epidemiological data on PND in rural areas across various countries and regions poses significant challenges to policy formulation and implementation. More research needs to focus on the level of maternal mental health in rural areas. Second, the prevalence data in this review show significant variation attributed to study design and measurement methods, which highlights the importance of high-quality study design and standardized measurement methods to provide more accurate data on PND in rural areas. Third, given the negative impact of PND on rural families and communities, measures to prevent PND in rural areas should focus on addressing determinants, such as strengthening the construction and planning of health service facilities, establishing an effective anti-violence legal system, enhancing gender equality and mental health education, fostering social connections, and providing economic support to rural communities. Finally, routine psychological assessment of pregnant women remains essential, notwithstanding the resource constraints typically encountered in rural areas. To address the accessibility of health services in rural areas, telehealth offers an option if broadband capacity is available [88]. Other approaches include engaging lay health workers through task-sharing [89] and integrating mental health services into primary care [90]. Countries should adopt a rational and effective approach to screening and managing PND in rural areas, according to their specific circumstances, to provide additional evidence-based support.

## 5. Conclusion

In summary, our systematic review and meta-analysis suggest that PND is prevalent among women in rural areas. Several risk factors including food insecurity have an impact on the prevalence of PND. There is a pressing need for increased attention to PND in rural settings across countries. In addition, targeted policies and interventions need to be developed to address disparities between urban and rural areas and to enhance the mental health and well-being of perinatal women in rural communities.

## Figures and Tables

**Table 1:** Subgroup analysis of prevalence of perinatal depression.

Subgroup	Number of studies	Prevalence of depressive symptoms (95% CI)	Weight	*p*-Value	*I* ^2^
Study design				<0.001	
Cross-sectional	68	24.2 (19.9–28.8)	67.1	—	98.6
Longitudinal	31	18.0 (14.4–22.0)	30.9	—	99.1
Randomized controlled trial	2	14.1 (13.2–14.9)	2.0	—	NA
Sampling method				<0.001	
Nonrandomized sampling	81	21.9 (18.4–25.5)	80.3	—	99.3
Randomized sampling	20	22.9 (16.6–29.8)	19.7	—	97.3
Setting				<0.001	
Community/primary health	56	21.5 (17.1–26.3)	55.7	—	99.2
Hospital-based	21	27.8 (21.9–34.2)	20.3	—	94.7
Population-based	18	15.1 (10.9–19.7)	18.1	—	99.2
Combination	6	32.6 (19.9–46.7)	5.8	—	96.7
Timepoint				<0.001	
Antenatal	43	23.3 (19.7–27.2)	42.8	—	98.7
Postnatal	53	21.1 (15.8–27.0)	52.2	—	99.4
Combined antenatal and postnatal	5	21.0 (14.5–28.4)	4.9	—	92.0
Country income group				<0.001	
Low	13	24.5 (18.3–31.3)	12.9	—	98.8
Lower middle	59	22.8 (18.2–27.8)	58.6	—	99.4
Upper middle	15	17.9 (14.7–21.3)	14.9	—	89.9
High	14	21.0 (14.2–28.7)	13.6	—	97.5
Assessment instrument				<0.001	
Self-reported screening instrument	87	22.8 (19.4–26.4)	86.2	—	99.3
Diagnostic interview	14	17.6 (12.7–23.1)	13.8	—	94.2
Self-reported instrument				<0.001	
EPDS	54	22.4 (19.2–25.8)	53.1	—	97.2
PHQ	12	17.9 (12.0–24.6)	12.1	—	99.5
BDI	6	36.1 (20.3–53.7)	6.0	—	99.4
SRQ	5	14.1 (7.0–23.2)	5.0	—	99.0
Others	10	28.2 (17.3–40.7)	10.0	—	98.8
Continent				<0.001	
Asia	63	21.2 (17.4–25.2)	62.6	—	98.6
Africa	23	25.3 (20.4–30.6)	22.8	—	99.3
Europe	5	22.0 (12.6–33.2)	4.9	—	95.2
North America	7	26.4 (19.2–34.1)	6.7	—	90.4
Oceania	3	9.4 (3.4–18.1)	3.0	—	NA
Country				<0.001	
India	23	19.4 (14.9–24.3)	22.7	—	95.1
Bangladesh	8	25.4 (17.6–34.2)	8.0	—	97.6
China	11	15.2 (13.3–17.1)	11.0	—	67.6
America	6	28.5 (21.2–36.5)	5.9	—	91.2
Ethiopia	7	26.7 (16.6–38.3)	6.9	—	98.8
Pakistan	5	34.6 (25.4–44.3)	5.0	—	97.5
Malawi	4	15.3 (7.0–26.1)	4.0	—	99.3
Ghana	3	10.1 (4.4–17.8)	3.0	—	NA
Iran	6	31.1 (15.7–49.0)	6.1	—	99.7
Nepal	3	11.7 (2.2–27.0)	2.9	—	NA
Others	25	23.7 (17.6–30.4)	24.3	—	98.1

Abbreviations: BDI, Beck Depression Inventory; EPDS, Edinburgh Postnatal Depression Scale; NA, not applicable; PHQ, patient health questionnaire; SRQ, self-report questionnaire.

**Table 2:** Meta-regression for the prevalence of perinatal depression.

Covariate	Meta-regression coefficient	95% CI	*p*-Value
Study design	−0.062	−0.117, −0.009	0.026
Sampling method	−0.008	−0.079, 0.064	0.836
Setting	−0.014	−0.042, 0.015	0.349
Gestational period	−0.030	−0.077, 0.017	0.212
Country income group	−0.028	−0.067, 0.010	0.153
Assessment instrument	−0.055	−0.135, 0.026	0.184
Continent	0.012	−0.020, 0.044	0.463

## Data Availability

Study data are available upon request from the corresponding author (Mei Sun) at smnjw2008@126.com.

## Supporting Information

Supporting Information S1: File 1 The supplementary file includes the search strategy.

Supporting Information S2: File 2 Basic characteristics of all included studies.

Supporting Information S3: File 3 PRISMA Checklist.
