# Supplementary material for: Global Prevalence of Perinatal Depression and Its Determinants Among Rural Women: A Systematic Review and Meta-Analysis
Source: Depress Anxiety. 2024 Sep 20;2024:1882604. doi: 10.1155/2024/1882604 (PMC11919136; doi:10.1155/2024/1882604)
Supplement: Supporting Information S1: File 1 — The supplementary file includes the search strategy. [file 1882604.f1.docx]

**STable 1: Search strategy (PubMed).**

| **#** | **Query** | **Results** |
| --- | --- | --- |
| 1 | "Depression"[Mesh] | 152,732 |
| 2 | "Depressive Disorder"[Mesh] | 122,723 |
| 3 | (Depressive Disorders) OR (Disorder, Depressive) OR (Neurosis, Depressive) OR (Disorders, Depressive) OR (Depressive Neuroses) OR (Depressive Neurosis) OR (Neuroses, Depressive) OR (Depression, Endogenous) OR (Depressions, Endogenous) OR (Endogenous Depression) OR (Endogenous Depressions) OR (Depressive Syndrome) OR (Depressive Syndromes) OR (Syndrome, Depressive) OR (Syndromes, Depressive) OR (Depressions, Neurotic) OR (Depression, Neurotic) OR (Neurotic Depression) OR (Neurotic Depressions) OR Melancholia OR Melancholias OR (Unipolar Depression) OR (Depression, Unipolar) OR (Depressions, Unipolar) OR (Unipolar Depressions) OR (Depressive Symptoms) OR (Depressive Symptom) OR (Symptom, Depressive) OR (Emotional Depression) OR (Depression, Emotional) OR Depress* | 660,282 |
| 4 | #1 OR #2 OR #3 | 660,282 |
| 5 | "Peripartum Period"[Mesh] | 1,778 |
| 6 | (Period, Peripartum) OR (Periods, Peripartum) OR (Peripartum Periods) OR Peripartum OR Peripartums OR (Peripartum Women) OR (Peripartum Womens) OR (Women, Peripartum) OR (Womens, Peripartum) OR postpartum OR postnatal OR pregnan∗ OR perinatal OR childbirth OR obstetr∗ OR labor OR labour OR puerperal OR parturition OR maternal OR puerper* OR post-birth OR (after birth) OR mother* OR intrapartum OR prenatal OR antenatal OR antepartum | 3,329,263 |
| 7 | #5 OR #6 | 3,329,263 |
| 8 | #4 AND #7 | 100,476 |
| 9 | "Depression, Postpartum"[Mesh] | 7,557 |
| 10 | (Postnatal Depression) OR (Depression, Postnatal) OR (Post-Partum Depression) OR (Depression, Post-Partum) OR (Post Partum Depression) OR (Postpartum Depression) OR (Post-Natal Depression) OR (Depression, Post-Natal) OR (Post Natal Depression) OR (Postnatal Dysphoria) OR (Dysphoria, Postnatal) OR (Post-Partum Dysphoria) OR (Dysphoria, Post-Partum) OR (Post Partum Dysphoria) OR (Postpartum Dysphoria) OR (Dysphoria, Postpartum) OR (Post-Natal Dysphoria) OR (Dysphoria, Post-Natal) OR (Post Natal Dysphoria) OR (Depression, Postpartum) | 16,671 |
| 11 | #9 OR #10 | 16,671 |
| 12 | #8 OR #11 | 100,568 |
| 13 | "Rural Population"[Mesh] | 69,554 |
| 14 | (Population, Rural) OR (Populations, Rural) OR (Rural Spatial Distribution) OR (Rural Populations) OR (Distribution, Rural Spatial) OR (Distributions, Rural Spatial) OR (Rural Spatial Distributions) OR (Rural Residence) OR (Residence, Rural) OR (Rural Residences) OR (Rural Communities) OR (Communities, Rural) OR (Community, Rural) OR (Rural Community) OR rural* OR remote* OR small-town OR outback OR farm* OR impoverish* OR poverty* OR tribal* OR underserv* OR (non metropolit*) OR suburb* OR village OR slum* OR hamlet OR dorp OR countryside OR rustic* | 816,261 |
| 15 | #13 OR #14 | 816,261 |
| 16 | #12 AND #15 | 5033 |

**STable 2: Search strategy (Web of Science).**

| **#** | **Query** | **Results** |
| --- | --- | --- |
| 1 | TS=(Depression OR Depressive Disorder OR Depress* OR Depressive Symptoms OR Depressive Symptom OR Symptom, Depressive OR Emotional Depression OR Depression, Emotional OR Depressive Disorders OR Disorder, Depressive OR Disorders, Depressive OR Neurosis, Depressive OR Depressive Neuroses OR Depressive Neurosis OR Neuroses, Depressive OR Depression, Endogenous OR Depressions, Endogenous OR Endogenous Depression OR Endogenous Depressions OR Depressive Syndrome OR Depressive Syndromes OR Syndrome, Depressive OR Syndromes, Depressive OR Depression, Neurotic OR Depressions, Neurotic OR Neurotic Depression OR Neurotic Depressions OR Melancholia OR Melancholias OR Depression, Unipolar OR Unipolar Depression OR Depressions, Unipolar OR Unipolar Depressions) | 1,092,841 |
| 2 | TS=(Peripartum Period OR Period, Peripartum OR Periods, Peripartum OR Peripartum Periods OR Peripartum OR Peripartums OR Peripartum Women OR Peripartum Womens OR Women, Peripartum OR Womens, Peripartum OR postpartum OR postnatal OR pregnan∗ OR perinatal OR childbirth OR obstetr∗ OR labor OR labour OR puerperal OR parturition OR maternal OR puerper* OR post-birth OR after birth OR mother* OR intrapartum OR prenatal OR antenatal OR antepartum) | 1,715,017 |
| 3 | #2 AND #1 | 78,038 |
| 4 | TS=(rural Population OR rural area OR Population, Rural OR Populations, Rural OR Rural Populations OR Rural Spatial Distribution OR Distribution, Rural Spatial OR Distributions, Rural Spatial OR Rural Residence OR Rural Spatial Distributions OR Residence, Rural OR Rural Residences OR Rural Communities OR Communities, Rural OR Community, Rural OR Rural Community OR rural* OR remote* OR small-town OR outback OR farm* OR impoverish* OR poverty* OR tribal* OR underserv* OR non metropolit* OR suburb* OR village OR slum* OR hamlet OR dorp OR countryside OR rustic*) | 1,695,391 |
| 5 | TS=(Postnatal Depression OR Depression, Postnatal OR Post-Partum Depression OR Depression, Post-Partum OR Post Partum Depression OR Postpartum Depression OR Post-Natal Depression OR Depression, Post-Natal OR Post Natal Depression OR Postnatal Dysphoria OR Dysphoria, Postnatal OR Post-Partum Dysphoria OR Dysphoria, Post-Partum OR Post Partum Dysphoria OR Postpartum Dysphoria OR Dysphoria, Postpartum OR Post-Natal Dysphoria OR Dysphoria, Post-Natal OR Post Natal Dysphoria OR Depression, Postpartum) | 27,397 |
| 6 | #5 OR #3 | 78,365 |
| **7** | #6 AND #4 | 4,433 |

**STable 3: Search strategy (Cochrane).**

| **#** | **Query** | **Results** |
| --- | --- | --- |
| 1 | MeSH descriptor: [Depression] explode all trees | 17,012 |
| 2 | MeSH descriptor: [Depressive Disorder] explode all trees | 15,183 |
| 3 | (Depressive Symptoms):ti,ab,kw OR (Depressive Symptom):ti,ab,kw OR (Symptom, Depressive):ti,ab,kw OR (Emotional Depression):ti,ab,kw OR (Depression, Emotional):ti,ab,kw OR (Depressive Disorders):ti,ab,kw OR (Disorder, Depressive):ti,ab,kw OR (Disorders, Depressive):ti,ab,kw OR (Neurosis, Depressive):ti,ab,kw OR (Depressive Neuroses):ti,ab,kw OR (Depressive Neurosis):ti,ab,kw OR (Neuroses, Depressive):ti,ab,kw OR (Depressions, Endogenous):ti,ab,kw OR (Depression, Endogenous):ti,ab,kw OR (Endogenous Depression):ti,ab,kw OR (Endogenous Depressions):ti,ab,kw OR (Depressive Syndrome):ti,ab,kw OR (Depressive Syndromes):ti,ab,kw OR (Syndrome, Depressive):ti,ab,kw OR (Syndromes, Depressive):ti,ab,kw OR (Depression, Neurotic):ti,ab,kw OR (Depressions, Neurotic):ti,ab,kw OR (Neurotic Depression):ti,ab,kw OR (Neurotic Depressions):ti,ab,kw OR (Melancholia):ti,ab,kw OR (Melancholias):ti,ab,kw OR (Unipolar Depression):ti,ab,kw OR (Depression, Unipolar):ti,ab,kw OR (Depressions, Unipolar):ti,ab,kw OR (Unipolar Depressions):ti,ab,kw OR (Depress*):ti,ab,kw | 110,360 |
| 4 | #1 OR #2 OR #3 | 110,434 |
| 5 | MeSH descriptor: [Peripartum Period] explode all trees | 39 |
| 6 | (Period, Peripartum):ti,ab,kw OR (Periods, Peripartum):ti,ab,kw OR (Peripartum Periods):ti,ab,kw OR (Peripartum):ti,ab,kw OR (Peripartums):ti,ab,kw OR (Peripartum Women):ti,ab,kw OR (Peripartum Womens):ti,ab,kw OR (Women, Peripartum):ti,ab,kw OR (Womens, Peripartum):ti,ab,kw OR (postpartum):ti,ab,kw OR (postnatal):ti,ab,kw OR (pregnan∗):ti,ab,kw OR (perinatal):ti,ab,kw OR (childbirth):ti,ab,kw OR (obstetr∗):ti,ab,kw OR (labor):ti,ab,kw OR (labour):ti,ab,kw OR (puerperal):ti,ab,kw OR (parturition):ti,ab,kw OR (maternal):ti,ab,kw OR (puerper*):ti,ab,kw OR (post-birth):ti,ab,kw OR (after birth):ti,ab,kw OR (mother*):ti,ab,kw OR (intrapartum):ti,ab,kw OR (prenatal):ti,ab,kw OR (antenatal):ti,ab,kw OR (antepartum):ti,ab,kw | 75.975 |
| 7 | #5 OR #6 | 75.975 |
| 8 | #4 AND #7 | 6.237 |
| 9 | MeSH descriptor: [Depression, Postpartum] explode all trees | 897 |
| 10 | (Postnatal Depression):ti,ab,kw OR (Depression, Postnatal):ti,ab,kw OR (Post-Partum Depression):ti,ab,kw OR (Depression, Post-Partum):ti,ab,kw OR (Post Partum Depression):ti,ab,kw OR (Postpartum Depression):ti,ab,kw OR (Post-Natal Depression):ti,ab,kw OR (Depression, Post-Natal):ti,ab,kw OR (Post Natal Depression):ti,ab,kw OR (Postnatal Dysphoria):ti,ab,kw OR (Dysphoria, Postnatal):ti,ab,kw OR (Post-Partum Dysphoria):ti,ab,kw OR (Dysphoria, Post-Partum):ti,ab,kw OR (Post Partum Dysphoria):ti,ab,kw OR (Postpartum Dysphoria):ti,ab,kw OR (Dysphoria, Postpartum):ti,ab,kw OR (Post-Natal Dysphoria):ti,ab,kw OR (Dysphoria, Post-Natal):ti,ab,kw OR (Post Natal Dysphoria):ti,ab,kw OR (Depression, Postpartum):ti,ab,kw | 3,405 |
| 11 | #9 OR #10 | 3,432 |
| 12 | MeSH descriptor: [Rural Population] explode all trees | 2,664 |
| 13 | (Population, Rural):ti,ab,kw OR (Populations, Rural):ti,ab,kw OR (Rural Spatial Distribution):ti,ab,kw OR (Rural Populations):ti,ab,kw OR (Distribution, Rural Spatial):ti,ab,kw OR (Distributions, Rural Spatial):ti,ab,kw OR (Rural Spatial Distributions):ti,ab,kw OR (Rural Residence):ti,ab,kw OR (Residence, Rural):ti,ab,kw OR (Rural Residences):ti,ab,kw OR (Rural Communities):ti,ab,kw OR (Communities, Rural):ti,ab,kw OR (Community, Rural):ti,ab,kw OR (Rural Community):ti,ab,kw OR (rural*):ti,ab,kw OR (remote*):ti,ab,kw OR (outback):ti,ab,kw OR (small-town):ti,ab,kw OR (farm*):ti,ab,kw OR (impoverish*):ti,ab,kw OR (tribal*):ti,ab,kw OR (poverty*):ti,ab,kw OR (underserv*):ti,ab,kw OR (non metropolit*):ti,ab,kw OR (suburb*):ti,ab,kw OR (village):ti,ab,kw OR (slum*):ti,ab,kw OR (hamlet):ti,ab,kw OR (dorp):ti,ab,kw OR (countryside):ti,ab,kw OR (rustic*):ti,ab,kw | 30,842 |
| 14 | #12 OR #13 | 30,876 |
| 15 | #8 OR #11 | 6,302 |
| 16 | #14 AND #15 | 497 |
| 17 | Trials | 430 |

**STable 4: Search strategy (Embase).**

| **#** | **Query** | **Results** |
| --- | --- | --- |
| 1 | 'depression'/exp | 692,978 |
| 2 | 'depressive symptoms':ab,ti OR 'depressive symptom':ab,ti OR 'symptom, depressive':ab,ti OR 'emotional depression':ab,ti OR 'depression, emotional':ab,ti OR 'depressive disorders':ab,ti OR 'disorder, depressive':ab,ti OR 'disorders, depressive':ab,ti OR 'neurosis, depressive':ab,ti OR 'depressive neuroses':ab,ti OR 'depressive neurosis':ab,ti OR 'neuroses, depressive':ab,ti OR 'depressions, endogenous':ab,ti OR 'depression, endogenous':ab,ti OR 'endogenous depression':ab,ti OR 'endogenous depressions':ab,ti OR 'depressive syndrome':ab,ti OR 'depressive syndromes':ab,ti OR 'syndrome, depressive':ab,ti OR 'syndromes, depressive':ab,ti OR 'depression, neurotic':ab,ti OR 'depressions, neurotic':ab,ti OR 'neurotic depression':ab,ti OR 'neurotic depressions':ab,ti OR 'melancholia':ab,ti OR 'melancholias':ab,ti OR 'unipolar depression':ab,ti OR 'depression, unipolar':ab,ti OR 'depressions, unipolar':ab,ti OR 'unipolar depressions':ab,ti OR 'depress*':ab,ti | 805,312 |
| 3 | 'perinatal period'/exp | 44,428 |
| 4 | 'period, peripartum':ab,ti OR 'periods, peripartum':ab,ti OR 'peripartum periods':ab,ti OR 'peripartum':ab,ti OR 'peripartums':ab,ti OR 'peripartum women':ab,ti OR 'peripartum womens':ab,ti OR 'women, peripartum':ab,ti OR 'womens, peripartum':ab,ti OR 'postpartum':ab,ti OR 'postnatal':ab,ti OR 'pregnan∗':ab,ti OR 'perinatal':ab,ti OR 'childbirth':ab,ti OR 'obstetr∗':ab,ti OR 'labor':ab,ti OR 'labour':ab,ti OR 'puerperal':ab,ti OR 'parturition':ab,ti OR 'maternal':ab,ti OR 'puerper*':ab,ti OR 'post-birth':ab,ti OR 'after birth':ab,ti OR 'mother*':ab,ti OR 'intrapartum':ab,ti OR 'prenatal':ab,ti OR 'antenatal':ab,ti OR 'antepartum':ab,ti | 1,188,130 |
| 5 | #1 OR #2 | 1,034,976 |
| 6 | #3 OR #4 | 1,192,562 |
| **7** | #5 AND #6 | 56,488 |
| 8 | 'postnatal depression'/exp | 16,912 |
| 9 | 'postnatal depression':ab,ti OR 'depression, postnatal':ab,ti OR 'post-partum depression':ab,ti OR 'depression, post-partum':ab,ti OR 'post partum depression':ab,ti OR 'postpartum depression':ab,ti OR 'post-natal depression':ab,ti OR 'depression, post-natal':ab,ti OR 'post natal depression':ab,ti OR 'postnatal dysphoria':ab,ti OR 'dysphoria, postnatal':ab,ti OR 'post-partum dysphoria':ab,ti OR 'dysphoria, post-partum':ab,ti OR 'post partum dysphoria':ab,ti OR 'postpartum dysphoria':ab,ti OR 'dysphoria, postpartum':ab,ti OR 'post-natal dysphoria':ab,ti OR 'dysphoria, post-natal':ab,ti OR 'post natal dysphoria':ab,ti OR 'depression, postpartum':ab,ti | 12,949 |
| 10 | #8 OR #9 | 20,175 |
| 11 | #7 OR #10 | 58,429 |
| 12 | 'rural population'/exp | 61,327 |
| 13 | 'rural area'/exp | 76,240 |
| 14 | 'population, rural':ab,ti OR 'populations, rural':ab,ti OR 'rural spatial distribution':ab,ti OR 'rural populations':ab,ti OR 'distribution, rural spatial':ab,ti OR 'distributions, rural spatial':ab,ti OR 'rural spatial distributions':ab,ti OR 'rural residence':ab,ti OR 'residence, rural':ab,ti OR 'rural residences':ab,ti OR 'rural communities':ab,ti OR 'communities, rural':ab,ti OR 'community, rural':ab,ti OR 'rural community':ab,ti OR 'rural*':ab,ti OR 'remote*':ab,ti OR 'outback':ab,ti OR 'small-town':ab,ti OR 'farm*':ab,ti OR 'impoverish*':ab,ti OR 'tribal*':ab,ti OR 'poverty*':ab,ti OR 'underserv*':ab,ti OR 'non metropolit*':ab,ti OR 'suburb*':ab,ti OR 'village':ab,ti OR 'slum*':ab,ti OR 'hamlet':ab,ti OR 'dorp':ab,ti OR 'countryside':ab,ti OR 'rustic*':ab,ti | 598,074 |
| 15 | #12 OR #13 OR #14 | 623,620 |
| 16 | #11 AND #15 | 1,967 |

**STable 5: Search strategy (CINAHL).**

| **S** | **Query** | **Results** |
| --- | --- | --- |
| 1 | (MH "Depression, Postpartum") OR ( Postnatal Depression OR Depression, Postnatal OR Post-Partum Depression OR Depression, Post-Partum OR Post Partum Depression OR Postpartum Depression OR Post-Natal Depression OR Depression, Post-Natal OR Post Natal Depression OR Postnatal Dysphoria OR Dysphoria, Postnatal OR Post-Partum Dysphoria OR Dysphoria, Post-Partum OR Post Partum Dysphoria OR Postpartum Dysphoria OR Dysphoria, Postpartum OR Post-Natal Dysphoria OR Dysphoria, Post-Natal OR Post Natal Dysphoria OR Depression, Postpartum ) | 9,321 |
| 2 | (MH "Depression+") OR ( Depressive Disorder OR Depress* OR Depressive Symptoms OR Depressive Symptom OR Symptom, Depressive OR Emotional Depression OR Depression, Emotional OR Depressive Disorders OR Disorder, Depressive OR Disorders, Depressive OR Neurosis, Depressive OR Depressive Neuroses OR Depressive Neurosis OR Neuroses, Depressive OR Depression, Endogenous OR Depressions, Endogenous OR Endogenous Depression OR Endogenous Depressions OR Depressive Syndrome OR Depressive Syndromes OR Syndrome, Depressive OR Syndromes, Depressive OR Depression, Neurotic OR Depressions, Neurotic OR Neurotic Depression OR Neurotic Depressions OR Melancholia OR Melancholias OR Depression, Unipolar OR Unipolar Depression OR Depressions, Unipolar OR Unipolar Depressions ) | 223,312 |
| 3 | (MH "Perinatal Period") OR ( Peripartum Period OR Period, Peripartum OR Periods, Peripartum OR Peripartum Periods OR Peripartum OR Peripartums OR Peripartum Women OR Peripartum Womens OR Women, Peripartum OR Womens, Peripartum OR postpartum OR postnatal OR pregnan∗ OR perinatal OR childbirth OR obstetr∗ OR labor OR labour OR puerperal OR parturition OR maternal OR puerper* OR post-birth OR after birth OR mother* OR intrapartum OR prenatal OR antenatal OR antepartum ) | 395,946 |
| 4 | S2 AND S3 | 22,759 |
| 5 | S1 OR S4 | 22,812 |
| 6 | (MH "Rural Population") OR ( Rural Area OR Population, Rural OR Populations, Rural OR Rural Populations OR Rural Spatial Distribution OR Distribution, Rural Spatial OR Distributions, Rural Spatial OR Rural Residence OR Rural Spatial Distributions OR Residence, Rural OR Rural Residences OR Rural Communities OR Communities, Rural OR Community, Rural OR Rural Community OR rural* OR remote* OR small-town OR outback OR farm* OR impoverish* OR poverty* OR tribal* OR underserv* OR non metropolit* OR suburb* OR village OR slum* OR hamlet OR dorp OR countryside OR rustic* ) | 171,691 |
| 7 | S5 AND S6 | 1,406 |

**STable 6: Search strategy (Ovid, including PsycINFO and JBI).**

| **S** | **Query** | **Results** |
| --- | --- | --- |
| 1 | (Depression OR Depressive Disorder OR Depress* OR Depressive Symptoms OR Depressive Symptom OR Symptom, Depressive OR Emotional Depression OR Depression, Emotional OR Depressive Disorders OR Disorder, Depressive OR Disorders, Depressive OR Neurosis, Depressive OR Depressive Neuroses OR Depressive Neurosis OR Neuroses, Depressive OR Depression, Endogenous OR Depressions, Endogenous OR Endogenous Depression OR Endogenous Depressions OR Depressive Syndrome OR Depressive Syndromes OR Syndrome, Depressive OR Syndromes, Depressive OR Depression, Neurotic OR Depressions, Neurotic OR Neurotic Depression OR Neurotic Depressions OR Melancholia OR Melancholias OR Depression, Unipolar OR Unipolar Depression OR Depressions, Unipolar OR Unipolar Depressions).ti,ab,kw. AND (Rural Population OR Rural Area OR Population, Rural OR Populations, Rural OR Rural Populations OR Rural Spatial Distribution OR Distribution, Rural Spatial OR Distributions, Rural Spatial OR Rural Residence OR Rural Spatial Distributions OR Residence, Rural OR Rural Residences OR Rural Communities OR Communities, Rural OR Community, Rural OR Rural Community OR rural* OR remote* OR small-town OR outback OR farm* OR impoverish* OR poverty* OR tribal* OR underserv* OR non metropolit* OR suburb* OR village OR slum* OR hamlet OR dorp OR countryside OR rustic*).ti,ab,kw. AND (Peripartum Period OR Period, Peripartum OR Periods, Peripartum OR Peripartum Periods OR Peripartum OR Peripartums OR Peripartum Women OR Peripartum Womens OR Women, Peripartum OR Womens, Peripartum OR postpartum OR postnatal OR pregnan* OR perinatal OR childbirth OR obstetr* OR labor OR labour OR puerperal OR parturition OR maternal OR puerper* OR post-birth OR after birth OR mother* OR intrapartum OR prenatal OR antenatal OR antepartum).ti,ab,kw. | 1,190 |

**STable 7: Search strategy (Scopus).**

| **S** | **Query** | **Results** |
| --- | --- | --- |
| 1 | ( TITLE-ABS-KEY ( "Depression" OR "Depressive Disorder" OR "Depress*" OR "Depressive Symptoms" OR "Depressive Symptom" OR "Symptom, Depressive" OR "Emotional Depression" OR "Depression, Emotional" OR "Depressive Disorders" OR "Disorder, Depressive" OR "Disorders, Depressive" OR "Neurosis, Depressive" OR "Depressive Neuroses" OR "Depressive Neurosis" OR "Neuroses, Depressive" OR "Depression, Endogenous" OR "Depressions, Endogenous" OR "Endogenous Depression" OR "Endogenous Depressions" OR "Depressive Syndrome" OR "Depressive Syndromes" OR "Syndrome, Depressive" OR "Syndromes, Depressive" OR "Depression, Neurotic" OR "Depressions, Neurotic" OR "Neurotic Depression" OR "Neurotic Depressions" OR "Melancholia" OR "Melancholias" OR "Depression, Unipolar" OR "Unipolar Depression" OR "Depressions, Unipolar" OR "Unipolar Depressions" ) AND TITLE-ABS-KEY ( "Peripartum Period" OR "Period, Peripartum" OR "Periods, Peripartum" OR "Peripartum Periods" OR "Peripartum" OR "Peripartums" OR "Peripartum Women" OR "Peripartum Womens" OR "Women, Peripartum" OR "Womens, Peripartum" OR "postpartum" OR "postnatal" OR "pregnan∗" OR "perinatal" OR "childbirth" OR "obstetr∗" OR "labor" OR "labour" OR "puerperal" OR "parturition" OR "maternal" OR "puerper*" OR "post-birth" OR "after birth" OR "mother*" OR "intrapartum" OR "prenatal" OR "antenatal" OR "antepartum" ) AND TITLE-ABS-KEY ( "rural Population" OR "rural area" OR "Population, Rural" OR "Populations, Rural" OR "Rural Populations" OR "Rural Spatial Distribution" OR "Distribution, Rural Spatial" OR "Distributions, Rural Spatial" OR "Rural Residence" OR "Rural Spatial Distributions" OR "Residence, Rural" OR "Rural Residences" OR "Rural Communities" OR "Communities, Rural" OR "Community, Rural" OR "Rural Community" OR "rural*" OR "remote*" OR "small-town" OR "outback" OR "farm*" OR "impoverish*" OR "poverty*" OR "tribal*" OR "underserv*" OR "non metropolit*" OR "suburb*" OR "village" OR "slum*" OR "hamlet" OR "dorp" OR "countryside" OR "rustic*" ) ) | 3,343 |
