# Supplementary material for: Global Prevalence of Perinatal Depression and Its Determinants Among Rural Women: A Systematic Review and Meta-Analysis
Source: Depress Anxiety. 2024 Sep 20;2024:1882604. doi: 10.1155/2024/1882604 (PMC11919136; doi:10.1155/2024/1882604)
Supplement: Supporting Information S2: File 2 — Basic characteristics of all included studies. [file 1882604.f2.docx]

**STable 1: Characteristics of** **studies included in the meta-analysis of the prevalence of perinatal depression.**

| **Study** | **Country** | **Setting** | **Design** | **Time point** | **Sampling method** | **Sample size** | **Identified case** | **Tools** | **Cut-off value** | **Rate** | **Quality score** |
| --- | --- | --- | --- | --- | --- | --- | --- | --- | --- | --- | --- |
| (Insan et al., 2023) | Bangladesh | Community | Cross-Sectional | Antenatal | Non-random | 235 | 133 | EPDS | ≥10 | 56.6% | 9 |
| (Shaun et al., 2022) | Bangladesh | Community | Cross-Sectional | Antenatal | Non-random | 481 | 43 | PHQ-9 | ≥10 | 8.94% | 8 |
| (Aslam et al., 2022) | India | Community | Cross-Sectional | Postpartum | Non-random | 134 | 13 | EPDS | ≥10 | 9.7% | 9 |
| (Raghavan et al., 2021) | India | Community | Cross-Sectional | Perinatal | Non-random | 564 | 135 | EPDS | ≥10 | 23.9% | 9 |
| (Maria et al., 2021) | India | Hospital | Cross-Sectional | Postpartum | Non-random | 231 | 29 | EPDS | ≥13 | 12.5% | 8 |
| (Jha et al., 2021) | India | Population-based | Cross-Sectional | Antenatal | Random | 457 | 13 | MINI | NA | 2.8% | 9 |
| (Joshi & Raut, 2019) | India | Hospital | Cross-Sectional | Postpartum | Non-random | 300 | 57 | EPDS | ≥10 | 19% | 7 |
| (Agarwala et al., 2019) | India | Community | Cross-Sectional | Postpartum | Random | 410 | 88 | EPDS | ≥10 | 21.5% | 7 |
| (Rathod et al., 2018) | India | Community | Cross-Sectional | Perinatal | Random | 224 | 20 | PHQ-9 | ≥10 | 8.9% | 8 |
|  |  | Primary health | Cross-Sectional | Perinatal | Random | 130 | 24 | PHQ-9 | ≥10 | 18.5% | 8 |
| (Shidhaye et al., 2017) | India | Primary health | Cross-Sectional | Antenatal | Non-random | 302 | 51 | EPDS | >12 | 16.9% | 10 |
| (George et al., 2016) | India | Community | Cross-Sectional | Antenatal | Random | 202 | 33 | CIS-R (ICD-10) | NA | 16.3% | 9 |
| (Srinivasan et al., 2015) | India | Hospital | Cross-Sectional | Antenatal | Non-random | 100 | 65 | EPDS | ≥13 | 65% | 8 |
| (Shivalli & Gururaj, 2015) | India | Hospital | Cross-Sectional | Postpartum | Random | 102 | 32 | EPDS | ≥13 | 31.4% | 11 |
| (Johnson et al., 2015) | India | Hospital | Cross-Sectional | Postpartum | Non-random | 123 | 56 | EPDS | ≥13 | 45.5% | 9 |
| (Shah et al., 2021) | India | Hospital | Cross-Sectional | Antenatal | Non-random | 200 | 83 | EPDS | ≥13 | 41.5% | 7 |
| (Raj et al., 2021) | India | Community | Cross-Sectional | Antenatal | Random | 220 | 60 | BDI Ⅱ | ≥14 | 27.3% | 7 |
| (Shriraam et al., 2019) | India | Population-based | Cross-Sectional | Postpartum | Non-random | 365 | 40 | EPDS | ≥10 | 11% | 6 |
| (Sudeepa et al., 2013) | India | Hospital | Cross-Sectional | Postpartum | Non-random | 244 | 28 | EPDS | ≥10 | 11.5% | 5 |
| (Zahidie et al., 2011) | Pakistan | Community | Cross-Sectional | Antenatal | Non-random | 375 | 229 | CES-D | ≥16 | 61.1% | 8 |
| (Anato et al., 2022) | Ethiopia | Community | Cross-Sectional | Postpartum | Random | 232 | 53 | EPDS | ≥13 | 22.8% | 7 |
| (Belay et al., 2019) | Ethiopia | Community | Cross-Sectional | Antenatal | Non-random | 304 | 25 | EPDS | ≥13 | 8.2% | 10 |
| (Mossie et al., 2017) | Ethiopia | Combination | Cross-Sectional | Antenatal | Random | 196 | 61 | BDI | ≥14 | 31.1% | 10 |
| (Wang et al., 2022) | China | Population-based | Cross-Sectional | Perinatal | Random sampling | Pre: 220  Post: 473 | Pre: 43  Post: 88 | EPDS | ≥9.5 | Pre: 19.5%  Post: 18.6% | 10 |
| (Chang et al., 2022) | China | Population-based | Cross-Sectional | Antenatal | Non-random | 1053 | 170 | DASS-C21 | ≥10 | 16.14% | 11 |
| (Jiang et al., 2021) | China | Population-based | Cross-Sectional | Perinatal | Non-random | Pre: 309  Post: 718 | Pre: 43  Post: 101 | DASS-C21 | ≥10 | Pre: 13.9%  Post: 14.1% | 8 |
| (Huang et al., 2021) | China | Population-based | Cross-Sectional | Perinatal | Random | Pre: 249  Post: 241 | Pre: 26  Post: 25 | EPDS | ≥9.5 | Pre: 10.4%  Post: 10.4% | 9 |
| (Wang et al., 2016) | China | Hospital | Cross-Sectional | Antenatal | Non-random | 396 | 55 | EPDS | ≥13 | 13.9% | 6 |
| (Wu et al., 2022) | China | Population-based | Cross-Sectional | Postpartum | Random | 1040 | 138 | EPDS | >10 | 13.3% | 8 |
| (Dingana et al., 2022) | Cameroon | Hospital | Cross-Sectional | Postpartum | Non-random | 207 | 66 | EPDS | >11 | 31.8% | 7 |
| (Mark et al., 2021) | Malawi | Primary health | Cross-Sectional | Postpartum | Non-random | 175 | 34 | SRQ | ≥8 | 19.4% | 7 |
| (Khan et al., 2021) | Pakistan | Primary health | Cross-Sectional | Antenatal | Random | 872 | 242 | PHQ-9 | ≥10 | 27.8% | 11 |
| (Atuhaire et al., 2021) | Uganda | Combination | Cross-Sectional | Postpartum | Random | 99 | 37 | Structured Clinical Interview (DSM-IV) | NA | 37.4% | 10 |
| (Lillie et al., 2020) | Ghana | Community | Cross-Sectional | Antenatal | Non-random | 374 | 74 | PHQ-9 | ≥10 | 19.8% | 9 |
| (Labrague et al., 2020) | Philippines | Combination | Cross-Sectional | Postpartum | Non-random | 165 | 27 | EPDS | >10 | 16.4% | 10 |
| (Ginja et al., 2020) | Britain | Population-based | Cross-Sectional | Perinatal | Non-random | 130 | 36 | EPDS | ≥10 | 27.7% | 9 |
| (Arachchi et al., 2019) | Sri Lanka | Hospital | Cross-Sectional | Antenatal | Non-random | 475 | 126 | EPDS | ≥10 | 26.5% | 8 |
| (January & Chimbari, 2018) | Zimbabwe | Primary health | Cross-Sectional | Postpartum | Non-random | 192 | 48 | Structured Clinical Interview (DSM-IV) | NA | 25% | 9 |
| (Stellenberg & Abrahams, 2015) | South Africa | Community | Cross-Sectional | Postpartum | Non-random | 159 | 80 | EPDS and BDI | Not reported | 50.3% | 6 |
| (Mohamed et al., 2014) | Egypt | Combination | Cross-Sectional | Postpartum | Non-random | 57 | 42 | EPDS | ≥13 | 73.7% | 6 |
| (Kakyo et al., 2012) | Uganda | Hospital | Cross-Sectional | Postpartum | Non-random | 202 | 87 | EPDS | ≥10 | 43.1% | 7 |
| (Ndokera & MacArthur, 2011) | Zambia | Hospital | Cross-Sectional | Postpartum | Non-random | 278 | 27 | SRQ | ≥8 | 9.7% | 6 |
| (Kozinszky et al., 2011) | Hungary | Primary health | Cross-Sectional | Postpartum | Non-random | 721 | 67 | LQ | ≥12 | 9.3% | 8 |
|  |  | Primary health | Cross-Sectional | Postpartum | Non-random | 579 | 115 | LQ | ≥12 | 19.9% | 8 |
| (Fisher et al., 2010) | Viet Nam | Community | Cross-Sectional | Perinatal | Non-random | Pre: 134  Post: 100 | Pre: 20  Post: 15 | SCID (DSM-IV) | NA | Pre: 14.9%  Post: 15.0% | 9 |
| (Ho-Yen et al., 2006) | Nepal | Primary health | Cross-Sectional | Postpartum | Non-random | 102 | 4 | EPDS | >12 | 3.9% | 9 |
| (Chaaya et al., 2002) | Lebanon | Hospital | Cross-Sectional | Postpartum | Non-random | 189 | 50 | EPDS | ≥13 | 26.5% | 7 |
| (Khadka et al., 2020) | Nepal | Community | Cross-Sectional | Postpartum | Random | 258 | 69 | PHQ-2 | ≥3 | 26.7% | 10 |
| (Dias et al., 2011) | Brazil | Community | Cross-Sectional | Antenatal | Non-random | 94 | 15 | MINI | NA | 16% | 7 |
| (Kheirabadi et al., 2009) | Iran | Primary health | Cross-Sectional | Postpartum | Non-random | 6627 | 3786 | BDI Ⅱ | ≥10 | 57.1% | 7 |
| (Kim & Dee, 2018) | America | Primary health | Cross-Sectional | Postpartum | Non-random | 223 | 95 | EPDS | ≥10 | 42.6% | 8 |
| (Jesse & Swanson, 2007) | America | Combination | Cross-Sectional | Antenatal | Non-random | 324 | 107 | BDI Ⅱ | ≥16 | 33% | 8 |
| (Richardson et al., 2012) | America | Hospital | Cross-Sectional | Antenatal | Non-random | 126 | 45 | EPDS | ≥11 | 35.7% | 6 |
| (Baker et al., 2005) | America | Community | Cross-Sectional | Postpartum | Non-random | 151 | 35 | PDSS | Not reported | 23.2% | 5 |
| (Nasreen et al., 2011) | Bangladesh | Community | Cross-Sectional | Antenatal | Non-random | 720 | 132 | EPDS | ≥10 | 18.3% | 9 |
| (Kabir et al., 2014) | Bangladesh | Community | Cross-Sectional | Postpartum | Non-random | 660 | 209 | EPDS | ≥10 | 31.7% | 10 |
| (Baker & Oswalt, 2008) | America | Community | Cross-Sectional | Postpartum | Non-random | 498 | 112 | PDSS | ≥69 | 22.5% | 5 |
| (Reddy et al., 2022) | India | Community | Cross-Sectional | Postpartum | Non-random | 229 | 65 | EPDS | ≥13 | 28.4% | 10 |
| (Vivilaki et al., 2021) | Greek | Hospital | Cross-Sectional | Postpartum | Non-random | 92 | 41 | EPDS | ≥9 | 44.6% | 9 |
| (Jarahi et al., 2015) | Iran | Primary health | Cross-Sectional | Antenatal | Non-random | 175 | 83 | BDI | ≥11 | 47.4% | 6 |
| (Sparling et al., 2020) | Bangladesh | Population-based | Cross-Sectional | Perinatal | Non-random | 1040 | 292 | EPDS | ≥10 | 28.1% | 8 |
| (Toyama et al., 2021) | Laos | Community | Cross-Sectional | Postpartum | Non-random | 120 | 21 | EPDS | ≥10 | 17.5% | 9 |
| (Tomita et al., 2023) | Nepal | Primary health | Cross-Sectional | Postpartum | Non-random | 299 | 25 | EPDS | >12 | 8.4% | 8 |
| (Takelle et al., 2023) | Ethiopia | Hospital | Cross-Sectional | Antenatal | Random | 53 | 37 | EPDS | ≥13 | 69.8% | 10 |
| (George & Johnson, 2022) | India | Primary health | Longitudinal | Antenatal and Postpartum | Random | 150 | Pre: 0  Post: 17 | Pre: CIS-R  Post: EPDS | Pre: NA Post:≥13 | Pre: 0  Post: 11.3% | 7 |
| (Shrestha et al., 2015) | India | Community | Longitudinal | Antenatal and Postpartum | Non-random | 200 | Pre: 20  Post: 24 | Structured Clinical Interview (ICD-10) | NA | Pre: 10.0%  Post: 12.0% | 6 |
| (Tsao et al., 2015) | China | Hospital | Longitudinal | Antenatal and Postpartum | Non-random | 162 | Pre: 28  Post: 39 | EPDS | ≥13 | Pre: 17.3%  Post: 24.1% | 6 |
| (Ross et al., 2011) | Canada | Hospital | Longitudinal | Antenatal and Postpartum | Non-random | Pre: 23  Post: 19 | Pre: 0  Post: 1 | EPDS | >12 | Pre: 0  Post: 5.3% | 6 |
| (Edhborg et al., 2011) | Bangladesh | Community | Longitudinal | Postpartum | Non-random | 674 | 95 | EPDS | ≥10 | 14.1% | 6 |
| (Gausia et al., 2009b) | Bangladesh | Community | Longitudinal | Postpartum | Non-random | 346 | 76 | EPDS | ≥10 | 22% | 7 |
| (Bilszta et al., 2008) | Australia | Community | Longitudinal | Antenatal and Postpartum | Non-random | 1058 | Pre: 36  Post: 90 | EPDS | Pre: ≥15  Post:≥13 | Pre: 3.4%  Post: 8.5% | 6 |
| (Rahman et al., 2003) | Pakistan | Community | Longitudinal | Antenatal and Postpartum | Non-random | Pre: 632  Post: 541 | Pre: 160  Post: 151 | SCAN (ICD-10) | NA | Pre: 25.3% Post: 28.0% | 8 |
| (Chandran et al., 2002) | India | Community | Longitudinal | Antenatal and Postpartum | Non-random | 359 | Pre: 58  Post: 71 | CIS-R (ICD-10) | NA | Pre: 16.2%  Post: 19.8% | 7 |
| (Catalao et al., 2023) | Malawi | Population-based | Longitudinal | Antenatal and Postpartum | Non-random | Pre: 3986  Post: 3976 | Pre: 907  Post: 285 | SRQ | ≥8 | Pre: 22.8%  Post: 7.2% | 7 |
| (Zarghami et al., 2019) | Iran | Primary health | Longitudinal | Antenatal and Postpartum | Non-random | 970 | Pre: 192  Post: 173 | EPDS | >12 | Pre: 19.8%  Post: 17.8% | 8 |
| (Bitew et al., 2019) | Ethiopia | Population-based | Longitudinal | Antenatal and Postpartum | Non-random | Pre: 1208  Post: 1142 | Pre: 371  Post: 340 | PHQ-9 | ≥5 | Pre: 30.7%  Post: 29.8% | 7 |
| (Weobong et al., 2015) | Ghana | Population-based | Longitudinal | Postpartum | Non-random | 9840 | 358 | PHQ-9 | Not reported | 3.6% | 6 |
| (Weobong et al., 2014) | Ghana | Population-based | Longitudinal | Antenatal | Non-random | 14400 | 1479 | PHQ-9 | Not reported | 10.3% | 6 |
| (Kheirabadi & Maracy, 2010) | Iran | Primary health | Longitudinal | Antenatal and Postpartum | Non-random | Pre: 1684 Post: 1291 | Pre: 384  Post: 340 | Pre: BDI Ⅱ  Post: EPDS | Pre: >20  Post:>12 | Pre: 22.8%  Post: 26.3% | 5 |
| (Dolbier et al., 2013) | America | Community | Longitudinal | Postpartum | Non-random | 299 | 52 | EPDS | ≥10 | 17.4% | 6 |
| (Mostafa et al., 2021) | Egypt | Primary health | Longitudinal | Antenatal | Random | 300 | 206 | HRSD | ≥8 | 68.7% | 8 |
| (Stewart et al., 2019) | Malawi | Combination | Longitudinal | Antenatal | Non-random | 1006 | 143 | SRQ | ≥8 | 14.2% | 7 |
| (Woldetensay et al., 2018) | Ethiopia | Community | Longitudinal | Antenatal | Non-random | 4680 | 506 | PHQ-9 | ≥8 | 10.81% | 6 |
| (Gausia et al., 2009a) | Bangladesh | Community | Longitudinal | Antenatal | Non-random | 361 | 119 | EPDS | ≥10 | 33.0% | 6 |
| (Galbally et al., 2022) | Australia | Hospital | Longitudinal | Antenatal | Non-random | 228 | 46 | SCID (DSM-IV) | NA | 20.2% | 7 |
| (Erten et al., 2022) | Turkey | Hospital | Longitudinal | Postpartum | Non-random | 178 | 31 | EPDS | ≥13 | 17.4% | 7 |
| (Matare et al., 2021) | Zimbabwe | Community | Randomized controlled trial | Antenatal | Non-random | 4574 | 395 | EPDS | ≥12 | 8.6% | 5 |
| (Sikander et al., 2019) | Pakistan | Primary health | Randomized controlled trial | Antenatal | Non-random | 1731 | 572 | PHQ-9 | ≥10 | 33.0% | 5 |

Note:

NA: not applicable; Pre: prenatal; Post: postnatal; EPDS: Edinburgh Postnatal Depression Scale; PHQ: Patient Health Questionnaire; BDI: Beck Depression Inventory; SRQ: Self Report Questionnaire; DASS: the Short Depression Anxiety and Stress Scale; LQ: Leverton 24-item questionnaire; PDSS: the Postpartum Depression Screening Scale; HRSD: the Hamilton Rating Scale for Depression; CES-D: Center for Epidemiological Studies-Depression; DSM: Diagnostic and Statistical Manual of Mental Disorders; ICD: International Classification of Diseases; SCID: Structured Clinical Interview for DSM; MINI: Mini-International Neuropsychiatric Interview; SCAN: Schedule for Clinical Assessment in Neuropsychiatry; CIS-R: Clinical Interview Schedule–Revised.

**References**

Agarwala, A., Rao, P. A., & Narayanan, P. (2019). Prevalence and predictors of postpartum depression among mothers in the rural areas of Udupi Taluk, Karnataka, India: A cross-sectional study [Article]. *Clinical Epidemiology and Global Health*, *7*(3), 342-345. <https://doi.org/10.1016/j.cegh.2018.08.009>

Anato, A., Baye, K., & Stoecker, B. (2022). Determinants of depressive symptoms among postpartum mothers: a cross-sectional study in Ethiopia. *BMJ Open*, *12*(9), e058633. <https://doi.org/10.1136/bmjopen-2021-058633>

Arachchi, N. S. M., Ganegama, R., Husna, A. W. F., Chandima, D. L., Hettigama, N., Premadasa, J., Herath, J., Ranaweera, H., Agampodi, T. C., & Agampodi, S. B. (2019). Suicidal ideation and intentional self-harm in pregnancy as a neglected agenda in maternal health; an experience from rural Sri Lanka. *Reprod Health*, *16*(1), 166. <https://doi.org/10.1186/s12978-019-0823-5>

Aslam, M., Nawab, T., Ahmad, A., Abedi, A. J., & Azmi, S. A. (2022). Postpartum Depression and its Clinico-Social Correlates-A Community-Based Study in Aligarh [Article]. *Indian Journal of Public Health*, *66*(4), 473-479. <https://doi.org/10.4103/ijph.ijph_1694_21>

Atuhaire, C., Rukundo, G. Z., Nambozi, G., Ngonzi, J., Atwine, D., Cumber, S. N., & Brennaman, L. (2021). Prevalence of postpartum depression and associated factors among women in Mbarara and Rwampara districts of south-western Uganda. *BMC Pregnancy Childbirth*, *21*(1), 503. <https://doi.org/10.1186/s12884-021-03967-3>

Baker, L., Cross, S., Greaver, L., Wei, G., & Lewis, R. (2005). Prevalence of postpartum depression in a native American population. *Matern Child Health J*, *9*(1), 21-25. <https://doi.org/10.1007/s10995-005-2448-2>

Baker, L., & Oswalt, K. (2008). Screening for postpartum depression in a rural community. *Community Ment Health J*, *44*(3), 171-180. <https://doi.org/10.1007/s10597-007-9115-6>

Belay, S., Astatkie, A., Emmelin, M., & Hinderaker, S. G. (2019). Intimate partner violence and maternal depression during pregnancy: A community-based cross-sectional study in Ethiopia. *PLoS One*, *14*(7), e0220003. <https://doi.org/10.1371/journal.pone.0220003>

Bilszta, J. L., Gu, Y. Z., Meyer, D., & Buist, A. E. (2008). A geographic comparison of the prevalence and risk factors for postnatal depression in an Australian population. *Aust N Z J Public Health*, *32*(5), 424-430. <https://doi.org/10.1111/j.1753-6405.2008.00274.x>

Bitew, T., Hanlon, C., Medhin, G., & Fekadu, A. (2019). Antenatal predictors of incident and persistent postnatal depressive symptoms in rural Ethiopia: a population-based prospective study. *Reprod Health*, *16*(1), 28. <https://doi.org/10.1186/s12978-019-0690-0>

Catalao, R., Chapota, H., Chorwe-Sungani, G., & Hall, J. (2023). The impact of depression at preconception on pregnancy planning and unmet need for contraception in the first postpartum year: a cohort study from rural Malawi. *Reprod Health*, *20*(1), 36. <https://doi.org/10.1186/s12978-023-01576-1>

Chaaya, M., Campbell, O. M., El Kak, F., Shaar, D., Harb, H., & Kaddour, A. (2002). Postpartum depression: prevalence and determinants in Lebanon. *Arch Womens Ment Health*, *5*(2), 65-72. <https://doi.org/10.1007/s00737-002-0140-8>

Chandran, M., Tharyan, P., Muliyil, J., & Abraham, S. (2002). Post-partum depression in a cohort of women from a rural area of Tamil Nadu, India. Incidence and risk factors. *Br J Psychiatry*, *181*, 499-504. <https://doi.org/10.1192/bjp.181.6.499>

Chang, F., Fan, X., Zhang, Y., Tang, B., & Jia, X. (2022). Prevalence of depressive symptoms and correlated factors among pregnant women during their second and third trimesters in northwest rural China: a cross-sectional study. *BMC Pregnancy Childbirth*, *22*(1), 38. <https://doi.org/10.1186/s12884-021-04340-0>

Dias, F. M. V., Junior, C. S. D., Franco, G. C., Teixeira, A. L., & Ribeiro, A. M. (2011). Pregnancy is associated with psychiatric symptoms in a low-income countryside community of Brazil [Article]. *Neuropsychiatric Disease and Treatment*, *7*, 709-714. <https://doi.org/10.2147/ndt.S26588>

Dingana, T. N., Ngasa, S. N., Ngasa, N. C., Sani, L. A., Abanda, C., Sanji, E. W., Bill, M., Niba, J. O., & Babila, C.-S. (2022). Prevalence and factors associated with post-partum depression in a rural area of Cameroon: a cross-sectional study [Article]. *Pan African Medical Journal*, *42*, Article 138. <https://doi.org/10.11604/pamj.2022.42.138.32347>

Dolbier, C., Rush, T., Sahadeo, L., Shaffer, M., & Thorp, J. (2013). Relationships of Race and Socioeconomic Status to Postpartum Depressive Symptoms in Rural African American and Non-Hispanic White Women. *Maternal & Child Health Journal*, *17*(7), 1277-1287. <https://doi.org/10.1007/s10995-012-1123-7>

Edhborg, M., Nasreen, H.-E., & Kabir, Z. (2011). Impact of postpartum depressive and anxiety symptoms on mothers' emotional tie to their infants 2-3 months postpartum: a population-based study from rural Bangladesh. *Archives of Women's Mental Health*, *14*(4), 307-316. <https://doi.org/10.1007/s00737-011-0221-7>

Erten, O., Biyik, I., Soysal, C., Ince, O., Keskin, N., & Tasci, Y. (2022). Effect of the Covid 19 pandemic on depression and mother-infant bonding in uninfected postpartum women in a rural region [Article]. *BMC Pregnancy and Childbirth*, *22*(1), Article 227. <https://doi.org/10.1186/s12884-022-04580-8>

Fisher, J., Thach, T., Buoi Thi, L., Kriitmaa, K., Rosenthal, D., & Tuan, T. (2010). Common perinatal mental disorders in northern Viet Nam: community prevalence and health care use [Article]. *Bulletin of the World Health Organization*, *88*(10), 737-745. <https://doi.org/10.2471/blt.09.067066>

Galbally, M., Watson, S. J., Coleman, M., Worley, P., Verrier, L., Padmanabhan, V., & Lewis, A. J. (2022). Rurality as a predictor of perinatal mental health and well-being in an Australian cohort. *Aust J Rural Health*. <https://doi.org/10.1111/ajr.12934>

Gausia, K., Fisher, C., Ali, M., & Oosthuizen, J. (2009a). Antenatal depression and suicidal ideation among rural Bangladeshi women: a community-based study. *Arch Womens Ment Health*, *12*(5), 351-358. <https://doi.org/10.1007/s00737-009-0080-7>

Gausia, K., Fisher, C., Ali, M., & Oosthuizen, J. (2009b). Magnitude and contributory factors of postnatal depression: a community-based cohort study from a rural subdistrict of Bangladesh. *Psychol Med*, *39*(6), 999-1007. <https://doi.org/10.1017/s0033291708004455>

George, C., Lalitha, A. R. N., Antony, A., Kumar, A. V., & Jacob, K. S. (2016). Antenatal depression in coastal South India: Prevalence and risk factors in the community [Article]. *International Journal of Social Psychiatry*, *62*(2), 141-147. <https://doi.org/10.1177/0020764015607919>

George, M., & Johnson, A. R. (2022). Incidence of Postpartum Depression and Its Association With Antenatal Psychiatric Symptoms: A Longitudinal Study in 25 Villages of Rural South Karnataka [Article]. *Indian Journal of Psychological Medicine*, *44*(1), 37-44. <https://doi.org/10.1177/0253717621991061>

Ginja, S., Jackson, K., Newham, J. J., Henderson, E. J., Smart, D., & Lingam, R. (2020). Rural-urban differences in the mental health of perinatal women: a UK-based cross-sectional study. *BMC Pregnancy Childbirth*, *20*(1), 464. <https://doi.org/10.1186/s12884-020-03132-2>

Ho-Yen, S. D., Bondevik, G. T., Eberhard-Gran, M., & Bjorvatn, B. (2006). The prevalence of depressive symptoms in the postnatal period in Lalitpur district, Nepal. *Acta Obstet Gynecol Scand*, *85*(10), 1186-1192. <https://doi.org/10.1080/00016340600753158>

Huang, Y., Liu, Y., Wang, Y., & Liu, D. (2021). Family function fully mediates the relationship between social support and perinatal depression in rural Southwest China. *BMC Psychiatry*, *21*(1), 151. <https://doi.org/10.1186/s12888-021-03155-9>

Insan, N., Forrest, S., Jaigirdar, A., Islam, R., & Rankin, J. (2023). Social Determinants and Prevalence of Antenatal Depression among Women in Rural Bangladesh: A Cross-Sectional Study. *Int J Environ Res Public Health*, *20*(3). <https://doi.org/10.3390/ijerph20032364>

January, J., & Chimbari, M. J. (2018). Prevalence and factors associated with postnatal depression among women in two rural districts of Manicaland, Zimbabwe. *S Afr J Psychiatr*, *24*, 1176. <https://doi.org/10.4102/sajpsychiatry.v24i0.1176>

Jarahi, L., Zavar, A., & Shahi, M. N. (2015). Evaluation of Depression and the Contributing Factors in Pregnant Women Referring to Urban and Rural Health Care Centers of Sarakhs City, Iran. *Journal of Midwifery & Reproductive Health*, *3*(2), 343-348. <https://search.ebscohost.com/login.aspx?direct=true&db=c8h&AN=120481588&site=ehost-live>

Jesse, D. E., & Swanson, M. S. (2007). Risks and resources associated with antepartum risk for depression among rural southern women. *Nurs Res*, *56*(6), 378-386. <https://doi.org/10.1097/01.Nnr.0000299856.98170.19>

Jha, S., Salve, H. R., Goswami, K., Sagar, R., & Kant, S. (2021). Prevalence of Common Mental Disorders among pregnant women-Evidence from population-based study in rural Haryana, India. *J Family Med Prim Care*, *10*(6), 2319-2324. <https://doi.org/10.4103/jfmpc.jfmpc_2485_20>

Jiang, Q., Guo, Y., Zhang, E., Cohen, N., Ohtori, M., Sun, A., Dill, S.-E., Singh, M. K., She, X., Medina, A., & Rozelle, S. D. (2021). Perinatal Mental Health Problems in Rural China: The Role of Social Factors [Article]. *Frontiers in Psychiatry*, *12*, Article 636875. <https://doi.org/10.3389/fpsyt.2021.636875>

Johnson, A. R., Edwin, S., Joachim, N., Mathew, G., Ajay, S., & Joseph, B. (2015). Postnatal depression among women availing maternal health services in a rural hospital in South India. *Pak J Med Sci*, *31*(2), 408-413. <https://doi.org/10.12669/pjms.312.6702>

Joshi, M. N., & Raut, A. V. (2019). Maternal depression and its association with responsive feeding and nutritional status of infants: A cross-sectional study from a rural medical college in central India. *J Postgrad Med*, *65*(4), 212-218. <https://doi.org/10.4103/jpgm.JPGM_479_18>

Kabir, Z. N., Hashima, E. N., & Edhborg, M. (2014). Intimate partner violence and its association with maternal depressive symptoms 6-8 months after childbirth in rural Bangladesh. *Global health action*, *7*, 1-7. <https://doi.org/10.3402/gha.v7.24725>

Kakyo, T. A., Muliira, J. K., Mbalinda, S. N., Kizza, I. B., & Muliira, R. S. (2012). Factors associated with depressive symptoms among postpartum mothers in a rural district in Uganda. *Midwifery*, *28*(3), 374-379. <https://doi.org/10.1016/j.midw.2011.05.001>

Khadka, R., Hong, S. A., & Chang, Y. S. (2020). Prevalence and determinants of poor sleep quality and depression among postpartum women: a community-based study in Ramechhap district, Nepal. *Int Health*, *12*(2), 125-131. <https://doi.org/10.1093/inthealth/ihz032>

Khan, R., Waqas, A., Mustehsan, Z. H., Khan, A. S., Sikander, S., Ahmad, I., Jamil, A., Sharif, M., Bilal, S., Zulfiqar, S., Bibi, A., & Rahman, A. (2021). Predictors of Prenatal Depression: A Cross-Sectional Study in Rural Pakistan. *Front Psychiatry*, *12*, 584287. <https://doi.org/10.3389/fpsyt.2021.584287>

Kheirabadi, G. R., & Maracy, M. R. (2010). Perinatal depression in a cohort study on Iranian women. *J Res Med Sci*, *15*(1), 41-49.

Kheirabadi, G. R., Maracy, M. R., Barekatain, M., Salehi, M., Sadri, G. H., Kelishadi, M., & Cassy, P. (2009). Risk factors of postpartum depression in rural areas of Isfahan Province, Iran. *Arch Iran Med*, *12*(5), 461-467.

Kim, Y., & Dee, V. (2018). Sociodemographic and Obstetric Factors Related to Symptoms of Postpartum Depression in Hispanic Women in Rural California. *J Obstet Gynecol Neonatal Nurs*, *47*(1), 23-31. <https://doi.org/10.1016/j.jogn.2017.11.012>

Kozinszky, Z., Dudas, R. B., Csatordai, S., Devosa, I., Tóth, E., Szabó, D., Sikovanyecz, J., Zádori, J., Barabás, K., & Pál, A. (2011). Social dynamics of postpartum depression: a population-based screening in South-Eastern Hungary. *Soc Psychiatry Psychiatr Epidemiol*, *46*(5), 413-423. <https://doi.org/10.1007/s00127-010-0206-2>

Labrague, L. J., McEnroe‐Petitte, D., Tsaras, K., Yboa, B. C., Rosales, R. A., Tizon, M. M., & D'Souza, M. S. (2020). Predictors of postpartum depression and the utilization of postpartum depression services in rural areas in the Philippines. *Perspectives in psychiatric care*, *56*(2), 308-315. <https://doi.org/10.1111/ppc.12428>

Lillie, M., Gallis, J. A., Hembling, J., Owusu, R. K., Ali, M., Abubakr-Bibilazu, S., Aborigo, R., Adam, H., McEwan, E., Awoonor-Williams, J. K., & Baumgartner, J. N. (2020). Prevalence and Correlates of Depression Among Pregnant Women Enrolled in a Maternal and Newborn Health Program in Rural Northern Ghana: a Cross-sectional Survey. *Glob Soc Welf*, *7*(2), 131-140. <https://doi.org/10.1007/s40609-020-00170-8>

Maria, C., Ramesh, N., Johnson, A. R., Prince, P. M., Rodrigues, A., Lekha, A., & Elias, A. (2021). Prevalence and determinants of postpartum anxiety among women availing health services at a rural maternity hospital in south india [Article]. *Journal of SAFOG*, *13*(1), 1-5. <https://doi.org/10.5005/jp-journals-10006-1858>

Mark, T. E., Latulipe, R. J., Anto-Ocrah, M., Mlongoti, G., Adler, D., & Lanning, J. W. (2021). Seasonality, Food Insecurity, and Clinical Depression in Post-Partum Women in a Rural Malawi Setting. *Matern Child Health J*, *25*(5), 751-758. <https://doi.org/10.1007/s10995-020-03045-8>

Matare, C. R., Mbuya, M. N. N., Dickin, K. L., Constas, M. A., Pelto, G., Chasekwa, B., Humphrey, J. H., & Stoltzfus, R. J. (2021). Maternal Capabilities Are Associated with Child Caregiving Behaviors Among Women in Rural Zimbabwe. *J Nutr*, *151*(3), 685-694. <https://doi.org/10.1093/jn/nxaa255>

Mohamed, H. A., Spencer, S. L., Al Swasy, A. H., Swidan, S. E., & Abouelenien, M. S. (2014). A social and Biological Approach for Postpartum Depression in Egypt [Article]. *Woman - Psychosomatic Gynaecology and Obstetrics*, *1*(C), 30-39. <https://doi.org/10.1016/j.woman.2014.10.002>

Mossie, T. B., Sibhatu, A. K., Dargie, A., & Ayele, A. D. (2017). Prevalence of Antenatal Depressive Symptoms and Associated Factors among Pregnant Women in Maichew, North Ethiopia: An Institution Based Study. *Ethiop J Health Sci*, *27*(1), 59-66. <https://doi.org/10.4314/ejhs.v27i1.8>

Mostafa, O. A., El-Rafie, M., Al Sayed, E. T., Khalil, M. A., & Zaki, S. M. (2021). Assessment of antepartum depression and its effect on pregnancy outcome in two primary health care units in qaliobia governorate, egypt [Article]. *Open Access Macedonian Journal of Medical Sciences*, *9*(E), 447-454. <https://doi.org/10.3889/oamjms.2021.5960>

Nasreen, H. E., Kabir, Z. N., Forsell, Y., & Edhborg, M. (2011). Prevalence and associated factors of depressive and anxiety symptoms during pregnancy: a population based study in rural Bangladesh. *BMC Womens Health*, *11*, 22. <https://doi.org/10.1186/1472-6874-11-22>

Ndokera, R., & MacArthur, C. (2011). The relationship between maternal depression and adverse infant health outcomes in Zambia: a cross-sectional feasibility study. *Child Care Health Dev*, *37*(1), 74-81. <https://doi.org/10.1111/j.1365-2214.2010.01129.x>

Raghavan, V., Khan, H. A., Seshu, U., Rai, S. P., Durairaj, J., Aarthi, G., Sangeetha, C., John, S., & Thara, R. (2021). Prevalence and risk factors of perinatal depression among women in rural Bihar: A community-based cross-sectional study. *Asian J Psychiatr*, *56*, 102552. <https://doi.org/10.1016/j.ajp.2021.102552>

Rahman, A., Iqbal, Z., & Harrington, R. (2003). Life events, social support and depression in childbirth: perspectives from a rural community in the developing world. *Psychol Med*, *33*(7), 1161-1167. <https://doi.org/10.1017/s0033291703008286>

Raj, K., Keshari, S. S., Shankar, H., & Kesarwani, P. (2021). Depression and its Association with Housing conditions and Family among Pregnant Women of Rural Varanasi [Article]. *Indian Journal of Community Health*, *33*(4), 575-579. <https://doi.org/10.47203/IJCH.2021.v33i04.006>

Rathod, S. D., Honikman, S., Hanlon, C., & Shidhaye, R. (2018). Characteristics of perinatal depression in rural central, India: a cross-sectional study. *Int J Ment Health Syst*, *12*, 68. <https://doi.org/10.1186/s13033-018-0248-5>

Reddy, D. T., Syed, I. A., Sharma, D., Pattnaik, S., Begum, J., & Ausvi, S. (2022). Prevalence of Postnatal Depression and Its Risk Factors among Postnatal Women in Rural Area of Srikakulam District. *National Journal of Community Medicine*, *11*(06), 262-266. <https://doi.org/10.5455/njcm.20200525111512>

Richardson, A., Field, T., Newton, R., & Bendell, D. (2012). Locus of control and prenatal depression. *Infant Behav Dev*, *35*(4), 662-668. <https://doi.org/10.1016/j.infbeh.2012.07.006>

Ross, L., Villegas, L., Dennis, C.-L., Bourgeault, I., Cairney, J., Grigoriadis, S., Steele, L., & Yudin, M. (2011). Rural residence and risk for perinatal depression: a Canadian pilot study. *Archives of Women's Mental Health*, *14*(3), 175-185. <https://doi.org/10.1007/s00737-011-0208-4>

Shah, A. K., John, S., Chawla, K., Vankar, G. K., & Jaiswal, A. (2021). Assessment of Association between Domestic Violence and Antenatal Depression in Rural Indian Population [Article]. *Journal of Clinical and Diagnostic Research*, *15*(9), VC11-VC15. <https://doi.org/10.7860/jcdr/2021/49204.15442>

Shaun, M. M. A., Nizum, M. W. R., Shuvo, M. A., Fayeza, F., Faruk, M. O., Alam, M. F., Ahmed, M. S., Zaman, S., Mali, S. K., & Hawlader, M. D. H. (2022). Association between depressive symptoms and poor sleep quality among pregnant women in Northern Rural Bangladesh: a community-based cross-sectional study [Article]. *BMC Psychiatry*, *22*(1), Article 201. <https://doi.org/10.1186/s12888-022-03839-w>

Shidhaye, P., Shidhaye, R., & Phalke, V. (2017). Association of gender disadvantage factors and gender preference with antenatal depression in women: a cross-sectional study from rural Maharashtra. *Soc Psychiatry Psychiatr Epidemiol*, *52*(6), 737-748. <https://doi.org/10.1007/s00127-017-1380-2>

Shivalli, S., & Gururaj, N. (2015). Postnatal depression among rural women in South India: do socio-demographic, obstetric and pregnancy outcome have a role to play? *PLoS One*, *10*(4), e0122079. <https://doi.org/10.1371/journal.pone.0122079>

Shrestha, N., Hazrah, P., & Sagar, R. (2015). Incidence and prevalence of postpartum depression in a rural community of India. *Journal of Chitwan Medical College*, *5*(2), 11-19.

Shriraam, V., Shah, P., Rani, M., & Sathiyasekaran, B. W. C. (2019). A community-based study of postpartum depression in rural Southern India. *Indian Journal of Social Psychiatry*, *35*(1). <https://doi.org/10.4103/ijsp.ijsp_13_18>

Sikander, S., Ahmad, I., Atif, N., Zaidi, A., Vanobberghen, F., Weiss, H. A., Nisar, A., Tabana, H., Ain, Q. U., Bibi, A., Bilal, S., Bibi, T., Liaqat, R., Sharif, M., Zulfiqar, S., Fuhr, D. C., Price, L. N., Patel, V., & Rahman, A. (2019). Delivering the Thinking Healthy Programme for perinatal depression through volunteer peers: a cluster randomised controlled trial in Pakistan. *Lancet Psychiatry*, *6*(2), 128-139. <https://doi.org/10.1016/s2215-0366(18)30467-x>

Sparling, T. M., Waid, J. L., Wendt, A. S., & Gabrysch, S. (2020). Depression among women of reproductive age in rural Bangladesh is linked to food security, diets and nutrition. *Public Health Nutr*, *23*(4), 660-673. <https://doi.org/10.1017/s1368980019003495>

Srinivasan, N., Murthy, S., Singh, A. K., Upadhyay, V., Mohan, S. K., & Joshi, A. (2015). Assessment of burden of depression during pregnancy among pregnant women residing in rural setting of chennai. *J Clin Diagn Res*, *9*(4), Lc08-12. <https://doi.org/10.7860/jcdr/2015/12380.5850>

Stellenberg, E. L., & Abrahams, J. M. (2015). Prevalence of and factors influencing postnatal depression in a rural community in South Africa. *Afr J Prim Health Care Fam Med*, *7*(1), 874. <https://doi.org/10.4102/phcfm.v7i1.874>

Stewart, R. C., Ashorn, P., Umar, E., Dewey, K. G., Ashorn, U., Creed, F., Rahman, A., Tomenson, B., Prado, E. L., & Maleta, K. (2019). Associations between antenatal depression and neonatal outcomes in Malawi. *Matern Child Nutr*, *15*(2), e12709. <https://doi.org/10.1111/mcn.12709>

Sudeepa, D., Madhukumar, S., & Gaikwad, V. (2013). A study on postnatal depression of women in rural Bangalore. *Int J Health Sci Res*, *3*(1), 1-6.

Takelle, G. M., Nakie, G., Rtbey, G., & Melkam, M. (2023). Depressive symptoms and associated factors among pregnant women attending antenatal care at Comprehensive Specialized Hospitals in Northwest Ethiopia, 2022: an institution-based cross-sectional study. *Front Psychiatry*, *14*, 1148638. <https://doi.org/10.3389/fpsyt.2023.1148638>

Tomita, Y., Kiriya, J., Silwal, R. C., Ong, K. I. C., Shibanuma, A., & Jimba, M. (2023). Association between the person-centered maternity care experience and mental health after delivery in urban and rural Dhading, Nepal: a cross-sectional study. *BMC Pregnancy Childbirth*, *23*(1), 398. <https://doi.org/10.1186/s12884-023-05709-z>

Toyama, N., Vongphoumy, I., Uehara, M., Sato, C., Nishimoto, F., Moji, K., Pongvongsa, T., Shirai, K., Takayama, T., Takahara, M., Tamashiro, Y., Endo, Y., Kounnavong, S., & Kobayashi, J. (2021). Impact of village health volunteer support on postnatal depressive symptoms in the remote rural areas of Lao People's Democratic Republic: a cross-sectional study. *Trop Med Health*, *49*(1), 28. <https://doi.org/10.1186/s41182-021-00316-0>

Tsao, Y., Creedy, D. K., & Gamble, J. (2015). Prevalence and psychological correlates of postnatal depression in rural Taiwanese women. *Health Care Women Int*, *36*(4), 457-474. <https://doi.org/10.1080/07399332.2014.946510>

Vivilaki, V., Charos, D., Maniatelli, E., Briana, D. D., & Lionis, C. (2021). Predictors of symptoms of depression during the early postpartum period in a sample of greek rural women [Article]. *Archives of Hellenic Medicine*, *38*(3), 360-366. <https://www.embase.com/search/results?subaction=viewrecord&id=L2007576581&from=export>

Wang, N., Mu, M., Liu, Z., Reheman, Z., Yang, J., Nie, W., Shi, Y., & Nie, J. (2022). Correlation between primary family caregiver identity and maternal depression risk in poor rural China. *Hong Kong Med J*, *28*(6), 457-465. <https://doi.org/10.12809/hkmj219875>

Wang, Y., Wang, X., Liu, F., Jiang, X., Xiao, Y., Dong, X., Kong, X., Yang, X., Tian, D., & Qu, Z. (2016). Negative Life Events and Antenatal Depression among Pregnant Women in Rural China: The Role of Negative Automatic Thoughts. *PLoS One*, *11*(12), e0167597. <https://doi.org/10.1371/journal.pone.0167597>

Weobong, B., Soremekun, S., ten Asbroek, A. H. A., Amenga-Etego, S., Danso, S., Owusu-Agyei, S., Prince, M., & Kirkwood, B. R. (2014). Prevalence and determinants of antenatal depression among pregnant women in a predominantly rural population in Ghana: The DON population-based study [Article]. *Journal of Affective Disorders*, *165*, 1-7. <https://doi.org/10.1016/j.jad.2014.04.009>

Weobong, B., Ten Asbroek, A. H. A., Soremekun, S., Danso, S., Owusu-Agyei, S., Prince, M., & Kirkwood, B. R. (2015). Determinants of postnatal depression in rural Ghana: Findings from the don population based cohort study [Article]. *Depression and Anxiety*, *32*(2), 108-119. <https://doi.org/10.1002/da.22218>

Woldetensay, Y. K., Belachew, T., Biesalski, H. K., Ghosh, S., Lacruz, M. E., Scherbaum, V., & Kantelhardt, E. J. (2018). The role of nutrition, intimate partner violence and social support in prenatal depressive symptoms in rural Ethiopia: community based birth cohort study. *BMC Pregnancy Childbirth*, *18*(1), 374. <https://doi.org/10.1186/s12884-018-2009-5>

Wu, Y., Ye, R., Wang, Q., Sun, C., Ji, Y., Zhou, H., & Chang, W. (2022). Association of COVID-19 Lockdown during the Perinatal Period with Postpartum Depression: Evidence from Rural Areas of Western China. *Health Commun*, *37*(12), 1488-1495. <https://doi.org/10.1080/10410236.2022.2036425>

Zahidie, A., Kazi, A., Fatmi, Z., Bhatti, M. T., & Dureshahwar, S. (2011). Social environment and depression among pregnant women in rural areas of Sind, Pakistan. *JPMA-Journal of the Pakistan Medical Association*, *61*(12), 1183.

Zarghami, M., Abdollahi, F., & Lye, M. S. (2019). A comparison of the prevalence and related risk factors for post-partum depression in Urban and Rural Areas [Article]. *Iranian Journal of Psychiatry and Behavioral Sciences*, *13*(2). <https://doi.org/10.5812/ijpbs.62558>
